# Morphogenesis and metabolomics reveal the compatible relationship among *Suillus bovinus*, *Phialocephala fortinii*, and their co-host, *Pinus massoniana*

**DOI:** 10.1128/spectrum.01453-23

**Published:** 2023-09-07

**Authors:** Xueguang Sun, Yanzhen Zhao, Guijie Ding

**Affiliations:** 1 Institute for Forest Resources & Environment of Guizhou, Guizhou University, Guiyang, China; 2 Key Laboratory of Forest Cultivation in Plateau Mountain of Guizhou Province, Guizhou University, Guiyang, Guizhou, China; 3 College of Forestry, Guizhou University, Guiyang, China; State Key Laboratory of Microbial Resources, Institute of Microbiology, Chinese Academy of Sciences, Beijing, China

**Keywords:** dark septate endophyte, ectomycorrhizal fungus, interactions, metabolome, *Pinus massoniana*

## Abstract

Ectomycorrhizal (ECM) fungi and dark septate endophytes (DSEs) can both form a symbiotic relationship with the same host plant. However, the interactions that occur among these two types of fungi and their co-hosts are largely unknown. Here, we investigated interactions that occur among the ECM fungus *Suillus bovinus*, the DSE *Phialocephala fortinii*, and their co-host *Pinus massoniana*. We used both scanning electron microscopy and optical microscopy to characterize the morphogenesis of the two symbionts and employed the ultra-high-performance liquid chromatography–tandem mass spectrometry technique to assess the effects of fungal inoculation on the root metabolome. Under pure culture conditions, no synergistic or antagonistic effects were observed between *Phi. fortinii* and *S. bovinus*. Generally, *S. bovinus* and *Phi. fortinii* can simultaneously colonize *P. massoniana* roots without affecting each other’s symbiotic processes. *S. bovinus* can colonize the root locus where *Phi. fortinii* has already invaded but not vice versa, which may be due to the physical barrier effect of the mantle. Both fungi can significantly promote the growth of *P. massoniana*, and they have a synergistic effect on host N and K uptake. Metabolite accumulation patterns in roots inoculated with *Phi. fortinii* and/or *S. bovinus* were greatly altered, especially with respect to organic acids, flavonoids, lipids, and phenolic acids. *S. bovinus* inoculation significantly enhanced root flavonoid biosynthesis, whereas *Phi. fortinii* and dual-inoculation treatments mainly induced phenylpropanoid biosynthesis. These findings reveal compatible relationships among *P. massoniana*, *S. bovinus*, and *Phi. fortinii*, and suggest a theoretical basis for ECM fungi and DSE co-application when cultivating seedlings.

**IMPORTANCE:**

The prevalence of both ectomycorrhizal fungi and dark septate endophytes in the roots of a wide spectrum of tree species is well recognized. In this study, we investigated the interactions that occur among the ECM fungus *S. bovinus*, the DSE *Phi. fortinii*, and their co-host, *P. massoniana*. The two fungi can simultaneously colonize *P. massoniana* roots without affecting each other’s symbiotic processes. *S. bovinus* appears to be superior to *Phi. fortinii* in microniche competition, which may be due to the physical barrier effect of the mantle. The two fungi have different effects on root metabolite accumulation patterns. *S. bovinus* inoculation significantly enhanced root flavonoid biosynthesis, whereas *Phi. fortinii* and dual-inoculation treatments mainly induced phenylpropanoid biosynthesis. This is the first study revealing the morphological and metabolic mechanisms that contribute to the compatible relationship among ECM fungi, DSEs, and their co-host.

## INTRODUCTION

Many dominant temperate tree species form associations with both ectomycorrhizal (ECM) fungi and dark septate endophytes (DSEs) (1 – 3), which can co-exist within the root system of the same host plant (4, 5). Both ECM fungi and DSE have been shown to promote the uptake of mineral nutrients by plants and to improve plant performance under abiotic and biotic stresses (2, 6, 7). Previous studies have tended to focus on the effect that an ECM fungus–host plant or DSE–host plant symbiosis has on the growth promotion and stress resistance of host plants (8 – 10). The interactions among DSE, ECM fungi, and their co-hosts have been less studied. Furthermore, the influence that DSE and ECM fungi have on each other’s growth and on the symbiotic process with the co-host plant, as well as the metabolic adjustments of plant roots to co-invasion by these two types of fungi, is less understood.

Interactions between ECM fungi and DSEs *in vitro* appear to be species- and strain-specific (4). Different combinations of fungi can produce positive, negative, or neutral interactions. ECM–host and DSE–host associations have distinct symbiotic structures. ECM fungi generally colonize host root tips, encasing both epidermal and cortical cells by forming an extraradical mantle and intraradical Hartig net, respectively (2, 11, 12). By contrast, DSEs are often found in lignified roots and are mainly characterized by darkly pigmented mycelium and intracellular microsclerotia, which form within host roots (2, 13). ECM fungi and DSEs appear to show spatial complementarity when colonizing host roots. However, it is not known whether the symbiotic processes of each of these two types of fungi have an impact on the formation of a symbiotic association by the other.

Several host transcriptional and metabolic pathways are impacted by ECM fungal colonization, although the pathways that are altered during colonization vary, depending on the plant/fungal species (11, 12, 14 – 17). Compared with ECM associations, molecular interactions between DSE and host plants have received much less attention (18). Differentially expressed genes detected in *Vaccinium corymbosum* colonized by *Anteaglonium* sp., a DSE, were associated with transcriptional regulation, material transport, phytohormone biosynthesis, and flavonoid biosynthesis (19). Furthermore, a DSE strain, S16, has been shown to promote many host metabolic pathways, including amino acid metabolism, the biosynthesis of other secondary metabolites, carbohydrate metabolism, energy metabolism, and lipid metabolism (20). We still do not know whether these transcriptional changes are species combination specific or common among DSE–host associations and if they also occur in ECM–host associations. Furthermore, whether there are interactions between ECM–host and DSE–host associations in the same host, in terms of transcriptional or metabolic characteristics, remains unclear.

*Pinus massoniana* Lamb. is one of the most widely distributed indigenous tree species in South China (21) and a host plant for both ECM fungi and DSE (22 – 24). In previous investigations, we found that *Suillus bovinus* (Sb) (L.: Fr.) Kuntze (23) and *Phialocephala fortinii* (Pf) Wang and Wilcox (22) are the dominant ECM fungus and DSE, respectively, associated with *P. massoniana*, and that both fungi promote *P. massoniana* growth (22 – 24). *S. bovinus* is ubiquitously distributed globally and shows a high degree of host specificity toward conifers (25 – 27). *Suillus* species (recognized as suilloid fungi) are the first colonizers of pine seedlings and play a vital role in conifer invasions (28, 29). *Phi. fortinii* belongs to the main representative of the DSE group—the *Phialocephala fortinii* s.l.–*Acephala applanata* species complex (PAC)—and is found in natural forest ecosystems in the Northern Hemisphere. Although *Phi. fortinii* is one of the most abundant DSEs and is known to dominate the endophytic mycobiota of conifer roots (18, 28, 30, 31), the interactions that occur among *S. bovinus*, *Phi. fortinii*, and their co-host, *P. massoniana*, have not been investigated.

In this study, we investigated the morphological and metabolic interactions that occur among *S. bovinus*, *Phi. fortinii*, and *P. massoniana*, aiming to elucidate the mechanism that contributes to the compatible relationship among them. We studied interactions between the two fungi under pure culture conditions and the effects that each fungal species has on each other’s symbiotic process with *P. massoniana*. The impacts of the fungal inoculation treatments, including the effects on the root metabolome, were also determined and compared.

## RESULTS

### Co-culture of *S. bovinus* and *Phi. fortinii*

**FIG 1:**
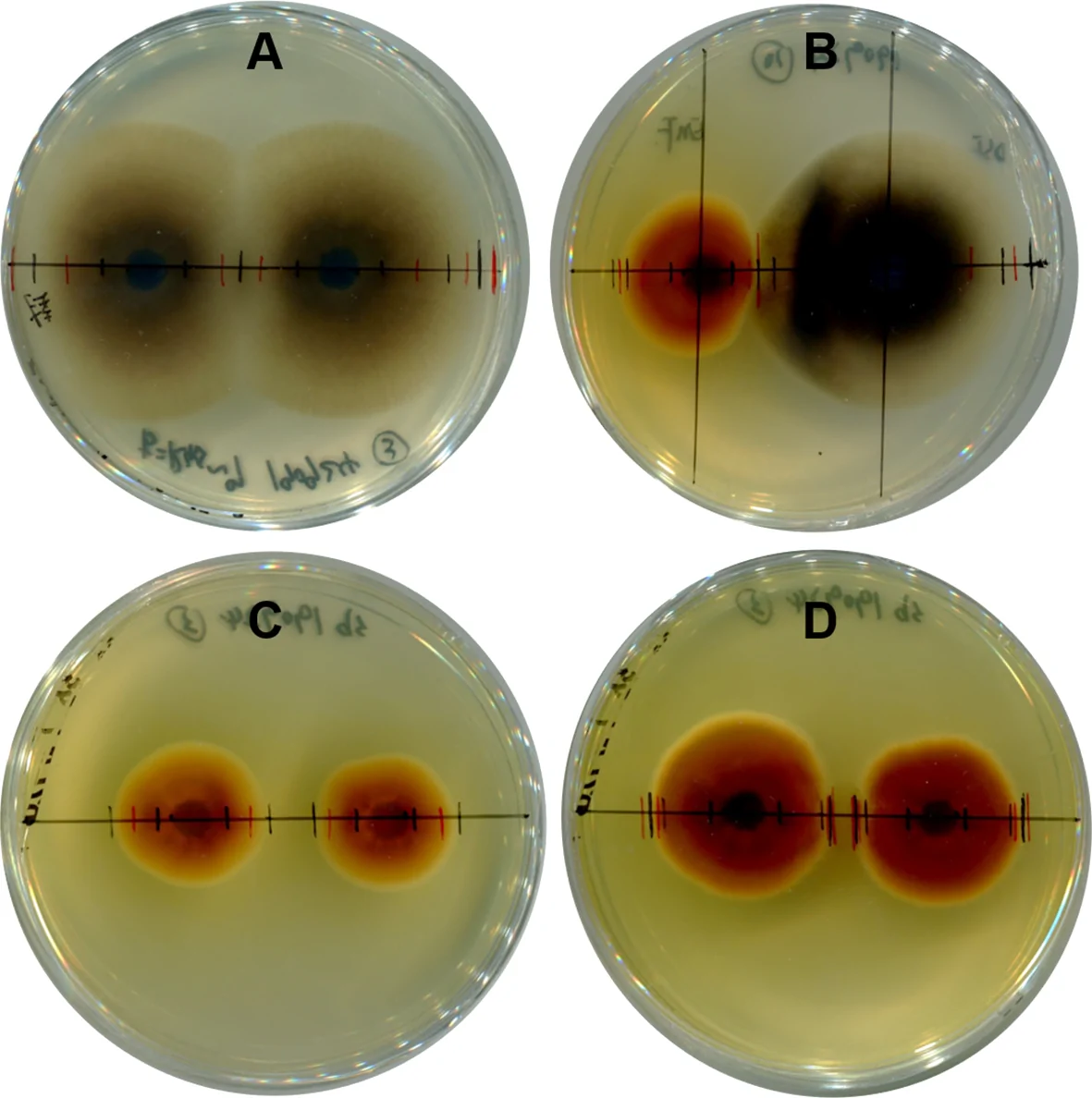
*Suillus bovinus* and *Phialocephala fortinii* colony morphology on potato dextrose agar medium. (**A**) Two colonies of *Phi. fortinii* at 20 dpi; (**B**) *S. bovinus* colony (left) and *Phi. fortinii* colony (right) at 20 dpi; two colonies of *S. bovinus* at 20 dpi (**C**) and 30 dpi (**D**).

*Phi. fortinii* grew faster than *S. bovinus* on potato dextrose agar (PDA) plates: the diameter of *Phi. fortinii* colonies (3.02 ± 0.14 cm) was significantly larger than those of *S. bovinus* (1.64 ± 0.16 cm) at 12 days postinoculation (dpi) (*P* < 0.05) (Table S1). Dual cultures of *Phi. fortinii* and *S. bovinus* showed no obvious promotion or inhibition effects when compared to plates inoculated with a single culture (controls) (Fig. 1; Table S1).

Melanin (indicated by the red elliptical ring in Fig. 1B) was synthetized by the *Phi. fortinii* colony when co-cultured with *S. bovinus*; however, this was not observed when *Phi. fortinii* was inoculated alone (Fig. 1A). In dual culture, the aerial mycelium (white and transparent) of *Phi. fortinii* was sparse in the interaction zone; however, the substrate mycelium (melanized septate hyphae) of *Phi. fortinii* was still growing densely under the substrate mycelium of *S. bovinus* (Fig. S1).

### Morphogenesis of symbioses formed by *S. bovinus* and *P. massoniana* or *Phi. fortinii* and *P. massoniana*

**FIG 2:**
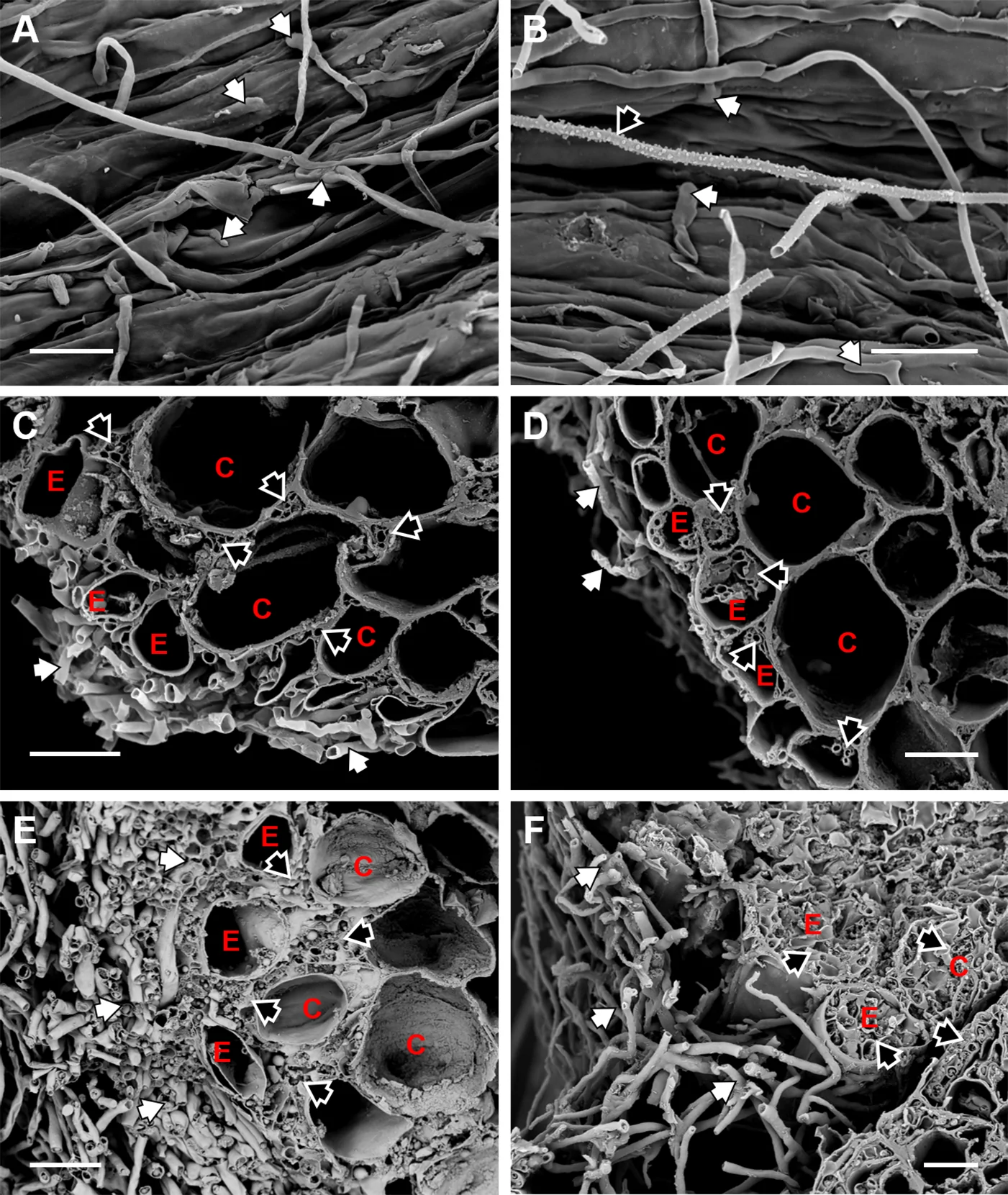
Symbiotic morphogenesis of *Suillus bovinus–Pinus massoniana* (**A, C, and E**), and *Phialocephala fortinii–P. massoniana* (**B, D, and F**). (**A**) *S. bovinus* mycelium attached to the root surface at 7 dpi. White arrowheads indicate swollen hyphal tips. (**B**) Extraradical mycelium of *Phi. fortinii* attached to the root surface at 2 dpi. White arrowheads indicate swollen hyphal tips; the black arrowhead indicates a granular hypha. (**C**) Intercellular (Hartig net at the early stages of development shown by black arrowheads) and extraradical (adhering to the root surface, indicated by white arrowheads) mycelium of *S. bovinus* at 14 dpi. (**D**) *Phi. fortinii* intracellular mycelium at 4 dpi (shown by black arrowheads) within root epidermal cells and extraradical mycelium (shown by white arrowheads) attached to the root surface. (**E**) Mantle (shown by white arrowheads) and Hartig net (shown by black arrowhead) formed by *S. bovinus* associated with a *P. massoniana* root at 28 dpi. (**F**) Extraradical mycelium (shown by white arrowheads) adhering to the root surface and intracellular mycelium filling root cells forming microsclerotia (shown by black arrowheads) at 8 dpi. Scale bars = 20 µm. C, cortical cell; E, epidermal cell.

The symbiotic association that formed between *Phi. fortinii* and *P. massoniana* developed faster than that between *S. bovinus* and *P. massoniana* (Fig. 2). *Phi. fortinii* formed microsclerotia in *P. massoniana* roots at 4 dpi, whereas the mantle and Hartig net formed by *S. bovinus* were observed at 28 dpi.

At 7 dpi, *S. bovinus* hyphae started attaching to the root surface (Fig. S2), and some hyphal tips swelled to form appressorium-like structures (Fig. 2A). At 14 dpi, the mantle and Hartig net started to develop in roots of all checked seedlings. A few hyphae penetrated the root and developed intercellularly, and meanwhile, the extraradical hyphae propagated and enveloped the root surface (Fig. 2C; see also Fig. S2C in the supplemental material). At 28 dpi, a fully developed Hartig net was observed surrounding both epidermal cells and cortical cells. A mantle comprising 7–12 layers of differentiated hyphae also formed (Fig. 2E; see also Fig. S2E; Fig. S3A and B in the supplemental material). Intracellular mycelium was absent.

At 2 dpi, *Phi. fortinii* hyphae began to attach to the root surface, and some hyphal tips swelled and formed appressorium-like structures (Fig. 2B; see also Fig. S2B in the supplemental material). At 4 dpi, both extraradical hyphae and intracellular hyphae were observed. Intracellular hyphae mainly colonized epidermal cells, forming clusters (an early form of microsclerotia) (Fig. 2D). Microsclerotia formed intensively in both epidermal and cortical cells at 8 dpi (Fig. 2F; see also Fig. S3C and D in the supplemental material), and some loosely arranged extraradical hyphae were also observed (Fig. 2F; see also Fig. S2F in the supplemental material). Intriguingly, the hyaline mycelium of *Phi. fortinii* became melanized when it attached to the root surface (Fig. S2D and F). Intercellular mycelium was less developed compared with intracellular mycelium, and the mycelium did not colonize the endodermis or stele cells.

### Impact of *S. bovinus* and *Phi. fortinii* co-inoculation on each other’s establishment of a symbiotic association with *P. massoniana*

**FIG 3:**
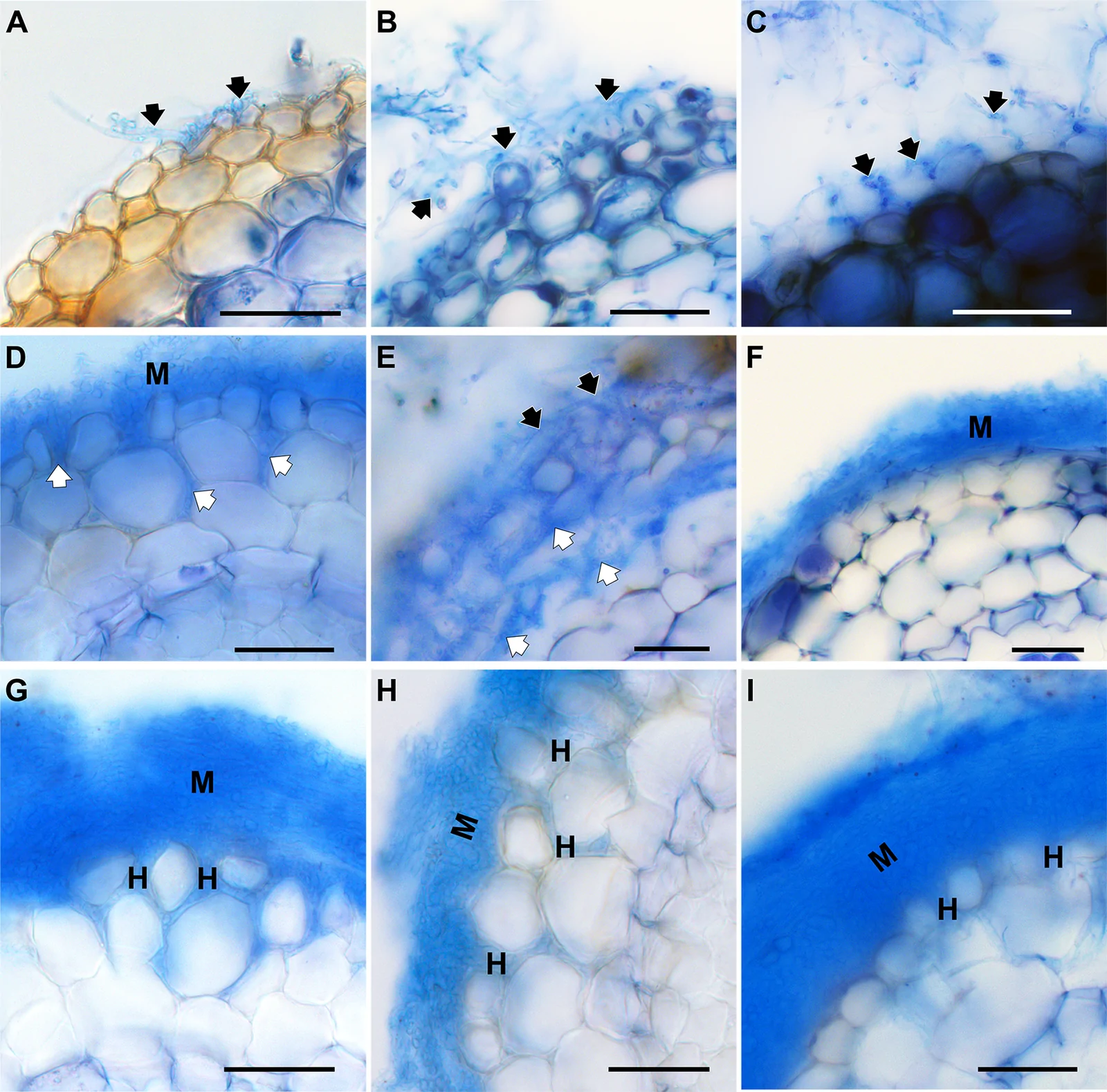
Symbiotic morphogenesis between *Suillus bovinus* (Sb) and *Pinus massoniana* roots. Roots had either been inoculated with Sb (**A, D, and G**); *Phi. fortinii* (Pf) followed 7 days later by *S. bovinus* inoculation (Pf + Sb) (**B, E, and H**), or simultaneously inoculated with both fungi (Sb–Pf) (**C, F, and I**). (**A through C**) Black arrowheads indicate extraradical mycelium, which is starting to invade the root at 7 dpi. Mycelium colonizing a root at 14 dpi and starting to form both a mantle (M) and a Hartig (H) net (**D**), an H net (**E**), and an M (**F**). White arrowheads indicate intracellular mycelium. (**G through I**) M and H net in seedling roots at 28 dpi. Scale bars = 50 µm.

In general, when seedlings were co-inoculated with *S. bovinus* and *Phi. fortinii* (i.e., Pf + Sb and Sb–Pf treatments), *Phi. fortinii* had little influence on the formation of a symbiotic association between *S. bovinus* and *P. massoniana* (Fig. 3). Mycelium started to attach to and invade the root at 7 dpi (Fig. 3A through C). The mantle and Hartig net began to develop at 14 dpi (Fig. 3D through F) and had fully formed by 28 dpi (Fig. 3G through I). However, mantle formation was slower (the extraradical hyphae loosely surrounding the root surface at 14 dpi) and thinner (five to seven layers of hyphae) in the Pf + Sb treatment than in the other treatments (Fig. 3H), and the Hartig net was less developed in the Sb–Pf treatment than in the other treatments (i.e., only surrounding the epidermal cells at 28 dpi) (Fig. 3I).

**FIG 4:**
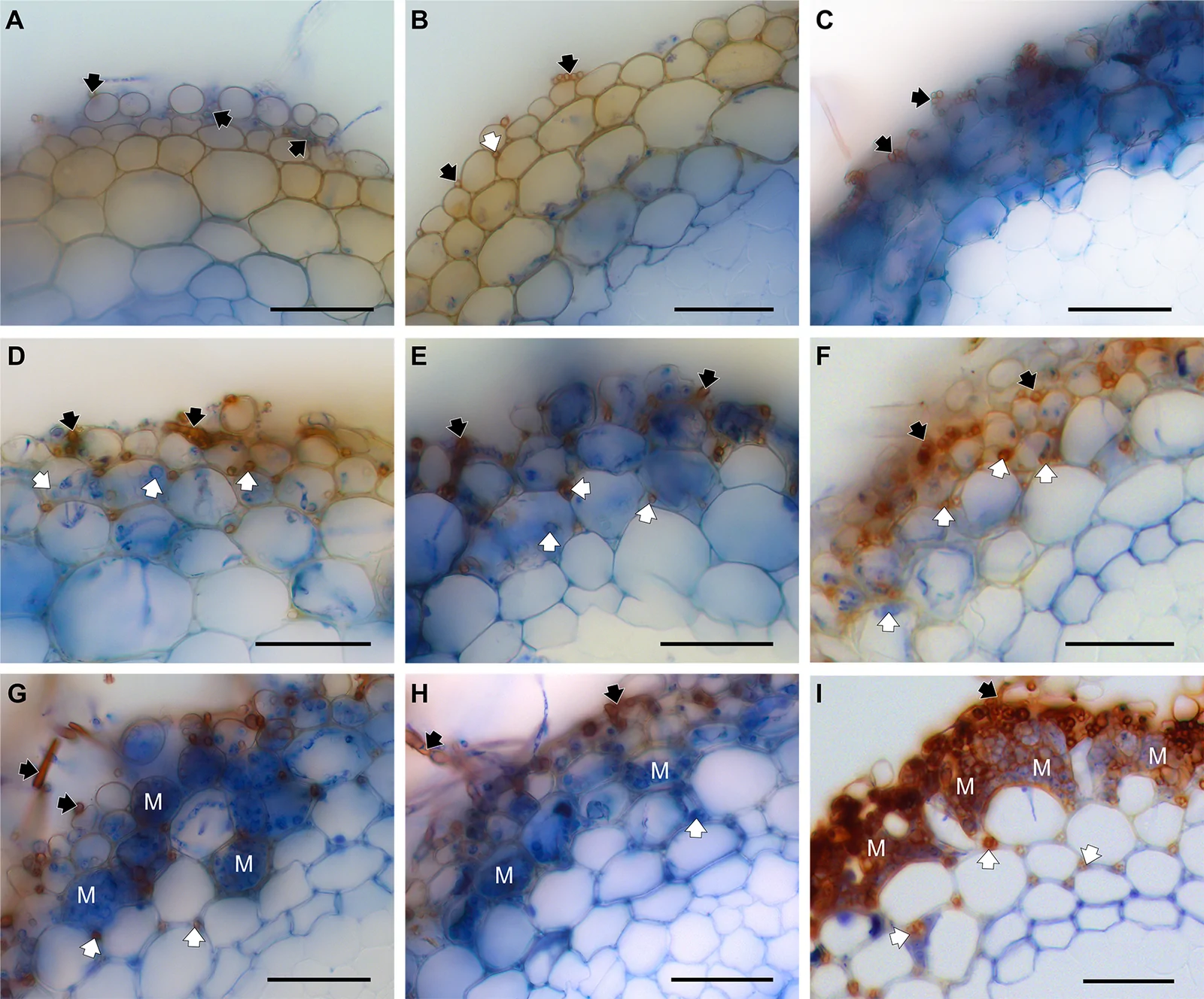
Symbiotic morphogenesis between *Phialocephala fortinii* and *Pinus massoniana* roots. Roots had either been inoculated with *Phi. fortinii* (Pf) (**A, D, and G**); *S. bovinus* (Sb) followed 28 days later by *Phi. fortinii* (Sb + Pf) (**B, E, and H**), or simultaneously inoculated with both fungi (Sb–Pf) (**C, F, and I**). (**A through C**) Black arrowheads indicate extraradical mycelium of *Phi. fortinii* attached to the root surface and the white arrowhead indicates intracellular mycelium that has started to invade the roots at 2 dpi. (**D through F**) *Phi. fortinii* extraradical mycelium and intracellular mycelium at 4 dpi. (**G through I**) Intensive formation of microsclerotia (M) within root cells at 28 dpi. Scale bars = 50 µm.

Similarly, when seedlings were co-inoculated with *Phi. fortinii* and *S. bovinus*, *S. bovinus* had a limited effect on the symbiotic process between *Phi. fortinii* and *P. massoniana* (Fig. 4). Mycelium attached to the root surface and started to colonize epidermal cells at 2 dpi (Fig. 4A through C). At 4 dpi, the mycelium proceeded to colonize the root and formed both intracellular (the prototype of microsclerotia) and intercellular structures (Fig. 4D through F). The formation of microsclerotia intensified within both epidermal and cortical cells at 8 dpi (Fig. 4G through I). Microsclerotia were mainly formed by hyaline mycelium in the Pf and Sb + Pf treatments (Fig. 4G and H), whereas in the Sb–Pf treatment, microsclerotia were formed by melanized mycelium (Fig. 4I).

**FIG 5:**
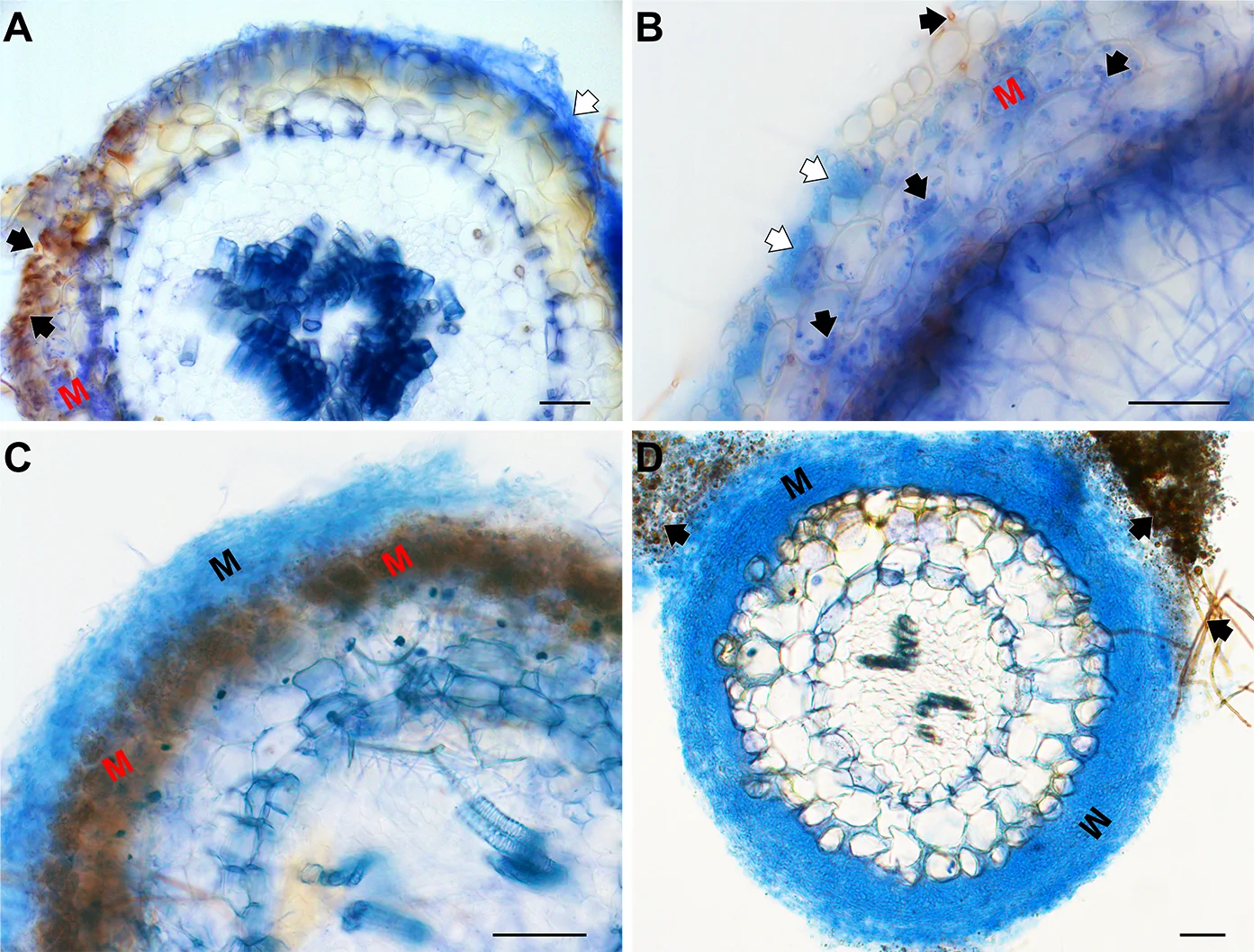
Colonization by both *Suillus bovinus* (Sb) and *Phialocephala fortinii* (Pf) at the same loci of a *Pinus massoniana* root that had been simultaneously inoculated with both fungi (Sb–Pf treatment) (28 dpi). (**A and B**) Black arrowheads indicate mycelium of *Phi. fortinii*, and white arrowheads indicate *S. bovinus* mycelium. Red M indicates microsclerotia. (**C**) Mantle (indicated by black M) formed by *S. bovinus* surrounding the root where *Phi. fortinii* has already colonized. (**D**) Black arrowheads indicate *Phi. fortinii* mycelium surrounding the mantle formed by *S. bovinus*. Scale bars = 50 µm.

*S. bovinus* and *Phi. fortinii* were able to simultaneously colonize the same root locus from different positions (Fig. 5A and B). Furthermore, *S. bovinus* was able to colonize the root locus and form a mantle-like structure at loci where *Phi. fortinii* had already formed intracellular microsclerotia (Fig. 5C). However, *Phi. fortinii* could not colonize a root locus if *S. bovinus* had already colonized and formed a mantle and Hartig net (Fig. 5D).

### Impact of *S. bovinus* and/or *Phi. fortinii* inoculation on *P. massoniana* seedling growth

**FIG 6:**
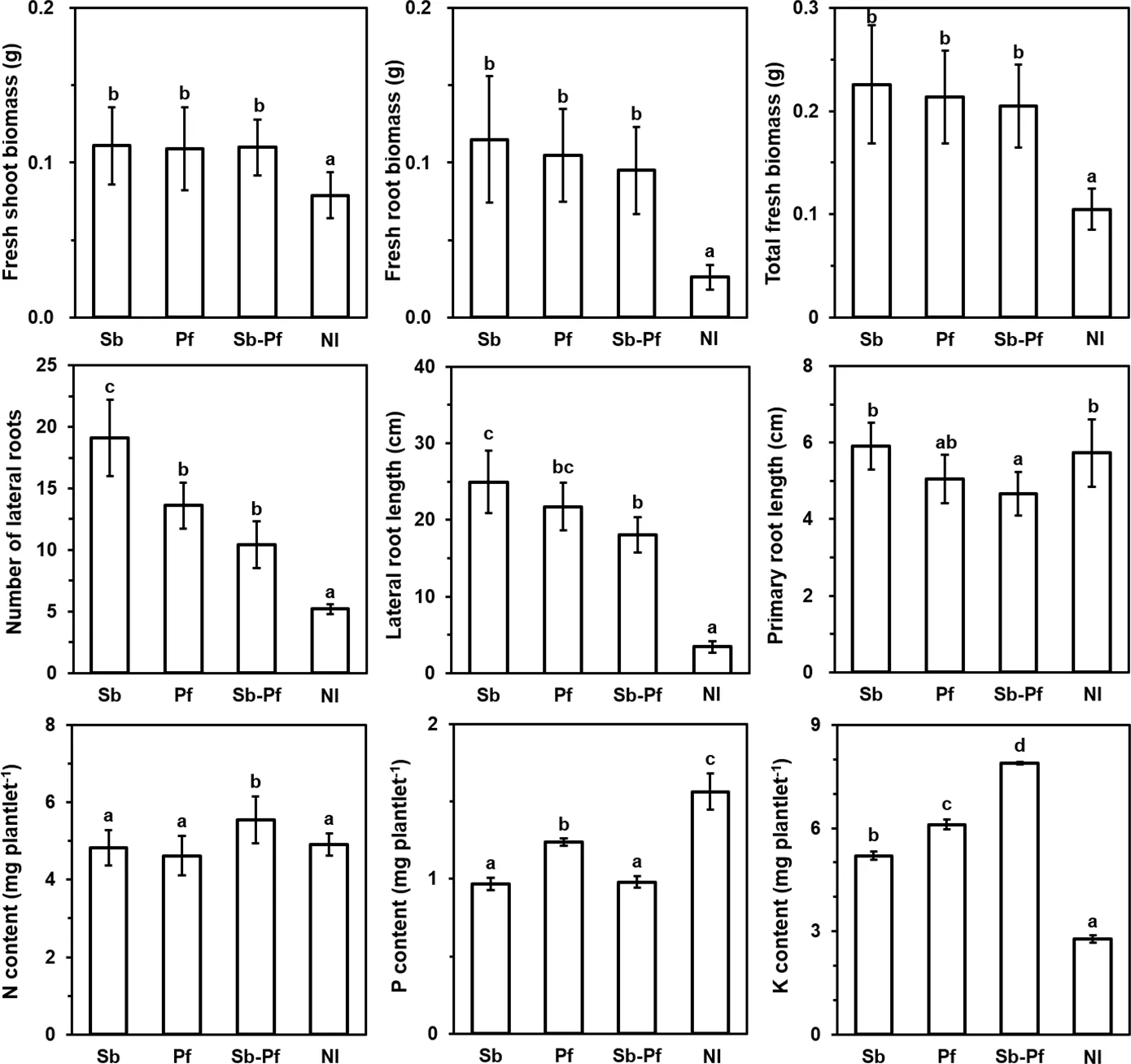
Biomass, root morphology, and nutrient content of *Pinus massoniana* seedlings. Different lowercase letters indicate significant differences in values between treatments (Student–Newman–Keuls *q* test was performed). *n* = 5. Treatment abbreviations: NI, seedlings that were not inoculated; Pf, seedlings inoculated with *Phialocephala fortinii*; Sb, seedlings inoculated with *Suillus bovinus*; Sb–Pf, seedlings inoculated with both fungi.

The inoculation of *S. bovinus* and/or *Phi. fortinii* significantly promoted *P. massoniana* lateral root ramification and growth, as well as root and shoot biomass accumulation (Fig. 6; see also Fig. S4 in the supplemental material) (*P* < 0.05). Furthermore, seedlings that received the Sb treatment developed significantly more lateral roots than the other seedlings. On average, 19.1 ± 3.1 lateral roots per seedling were formed in the Sb treatment, and it was 13.6 ± 1.9, 10.4 ± 1.9, and 5.2 ± 0.4 for that of Pf, Sb-Pf, and seedlings that were not inoculated (NI) treatments, respectively. However, dual inoculation of *S. bovinus* and *Phi. fortinii* (Sb–Pf treatment) inhibited primary root elongation (4.67 ± 0.57 cm compared with 5.73 ± 0.88 cm of NI treatment, *P* < 0.05).

Fungal inoculation also altered the nutrient content of *P. massoniana* seedlings (Fig. 6). Although single inoculation with either *S. bovinus* or *Phi. fortinii* had no effect on N accumulation, dual inoculation significantly increased N accumulation (*P* < 0.05). Astonishingly, fungal inoculation hampered *P* accumulation but greatly promoted K accumulation (*P* < 0.05) (Fig. 6). Dual inoculation also had a synergistic effect on host K uptake (2.8 times higher than that of NI treatment).

### Metabolic adjustments of *P. massoniana* roots following inoculation with *S. bovinus* and/or *Phi. fortinii*

**Fig 7:**
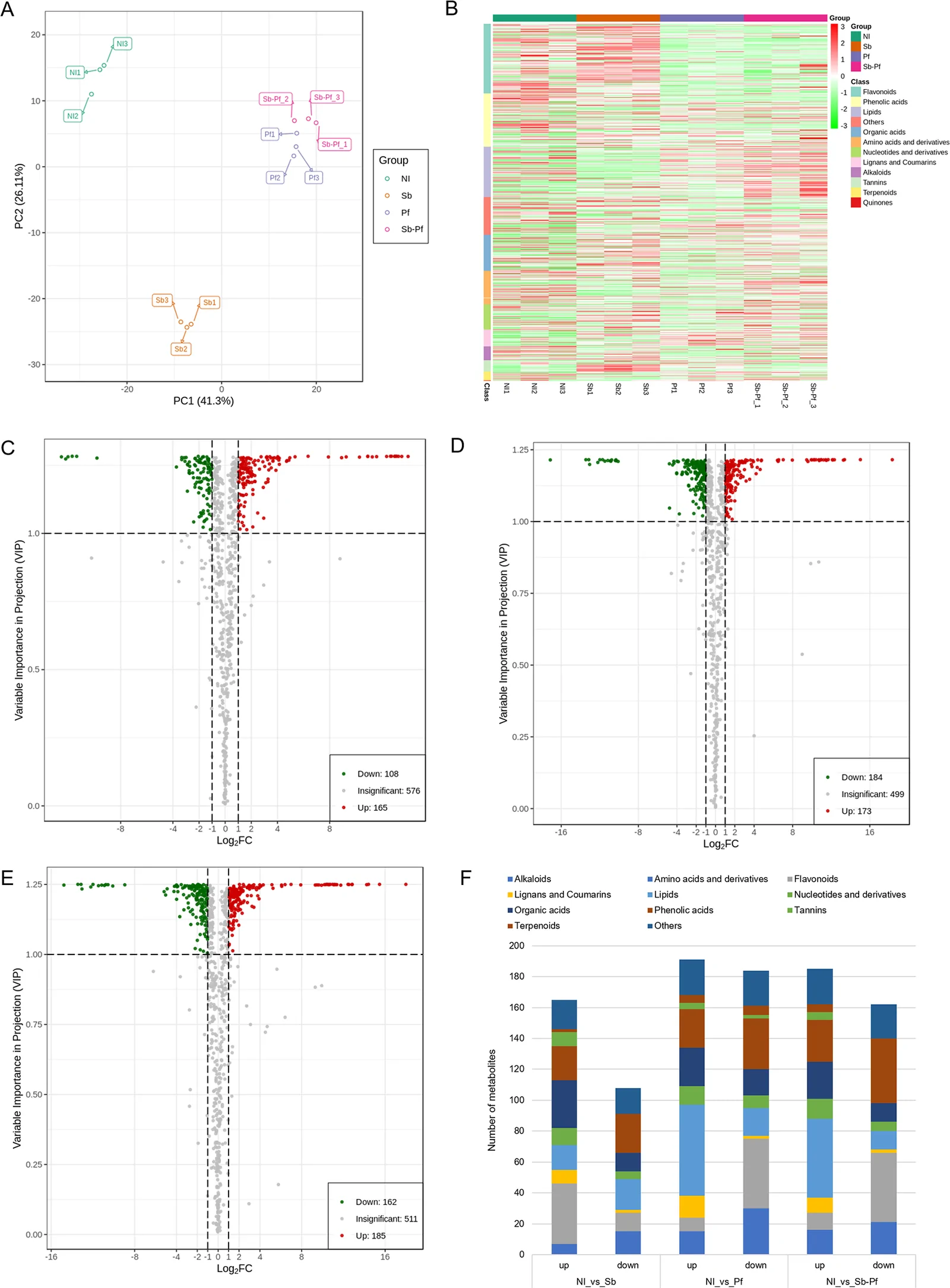
Overview of widely targeted metabolome analysis of *Pinus massoniana* roots. (**A**) Principal component analysis of metabolites. (**B**) Heatmap visualization of metabolites. The content of each metabolite was normalized to complete linkage hierarchical clustering. Each example was visualized in a single column, and each metabolite is represented by a single row. Red indicates high abundance, whereas green indicates metabolites with low relative abundance. (**C through E**) Volcano plots showing the number of differentially accumulated metabolites (DAMs) in the roots of seedlings inoculated with *Suillus bovinus* (Sb) vs NI, seedlings inoculated with *Phialocephala fortinii* (Pf) vs NI, and seedlings inoculated with both fungi (Sb–Pf) vs NI. (**F**) DAM categories.

Metabolomic analysis of *P. massoniana* roots following inoculation with *S. bovinus* and/or *Phi. fortinii* identified 861 metabolites, which were further categorized into 12 classes (Fig. 7; Table S2). Flavonoids (168 metabolites), phenolic acids (129), and lipids (121) were the most abundant metabolites.

Principal component analysis (PCA) showed that the first two principal components (PC1 and PC2) accounted for more than 67% of the variation in metabolic composition among samples. Most samples subjected to the same treatment aggregated together and separated from other treatments, revealing that different types of samples showed distinct accumulation patterns (Fig. 7A). In contrast, clusters of Pf and Sb–Pf samples did not form distinct groups, indicating that their metabolite accumulation patterns were similar. K-means analysis supported the PCA, and subclasses were selected as marker metabolites to discriminate between two different types of sample (e.g., subclass 3 could be used to distinguish NI and Sb treatments) (Fig. S5).

Inoculation with *S. bovinus* and/or *Phi. fortinii* greatly altered metabolite accumulation in *P. massoniana* roots (Fig. 7; Table S3). Compared with the NI treatment, 273, 357, and 347 differentially accumulated metabolites (DAMs) were detected in seedling roots subjected to the Sb, Pf, or Sb–Pf treatments, respectively. The most common types of DAMs detected in seedlings subjected to the Sb treatment were flavonoids (with 39 upregulated and 12 downregulated), organic acids (with 31 upregulated and 12 downregulated), and phenolic acids (22 upregulated and 17 downregulated). Under the Pf treatment, most DAMs were either lipids (59 upregulated and 18 downregulated), phenolic acids (25 upregulated and 33 regulated), or flavonoids (9 upregulated and 45 downregulated). Likewise, under the Sb–Pf treatment, most DAMs were either phenolic acids (27 upregulated and 37 downregulated), lipids (51 upregulated and 12 downregulated), or flavonoids (11 upregulated and 45 downregulated).

**Fig 8:**
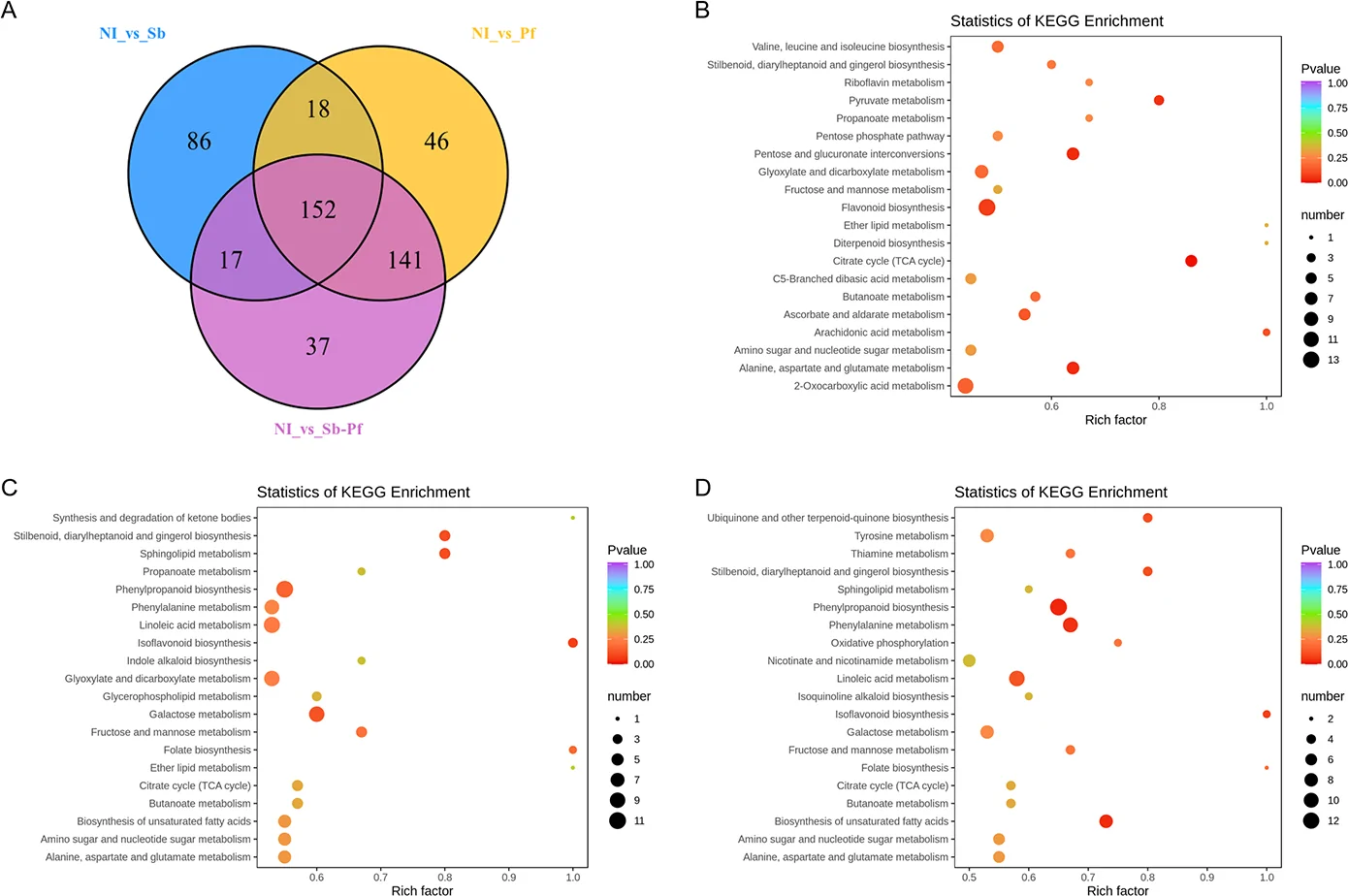
Venn diagram and pathway analysis of differentially accumulated metabolites detected in the roots of *Pinus massoniana* seedlings for three comparison groups. The comparison groups are seedlings that were not inoculated vs seedlings inoculated with *Suillus bovinus* (Sb), NI vs seedlings that were inoculated with *Phialocephala fortinii* (Pf), and NI vs seedlings inoculated with both fungi (Sb–Pf). (**A**) Venn diagram shows the overlapping and unique metabolites among the comparison groups. (**B through D**) KEGG (Kyoto Encyclopedia of Genes and Genomes) enrichment of differential metabolites between the comparison groups. Each bubble in the plot represents a metabolic pathway. The abscissa and bubble size jointly indicate the magnitude of the impact factors of the pathway. A larger bubble indicates a larger impact factor. Bubble colors represent the *P* values of the enrichment analysis, with darker colors showing a higher degree of enrichment.

Among all the detected DAMs (as compared with those metabolites in the NI treatment), 152 DAMs were detected in all the roots subjected to a fungal inoculation treatment (Fig. 8) and were mostly organic acids (24), lipids (23), or phenolic acids (23). Like the PCA, the DAM patterns of seedlings subjected to Pf or Sb–Pf treatments were more similar (they shared 293 DAMs) than under other treatments. In particular, methyl jasmonate levels were significantly higher in roots subjected to Pf or Sb–Pf treatments than in those subjected to NI or Sb treatments. Under the Sb treatment, 86 unique DAMs were detected, which were mostly flavonoids (24), organic acids (15), or phenolic acids (14).

**Fig 9:**
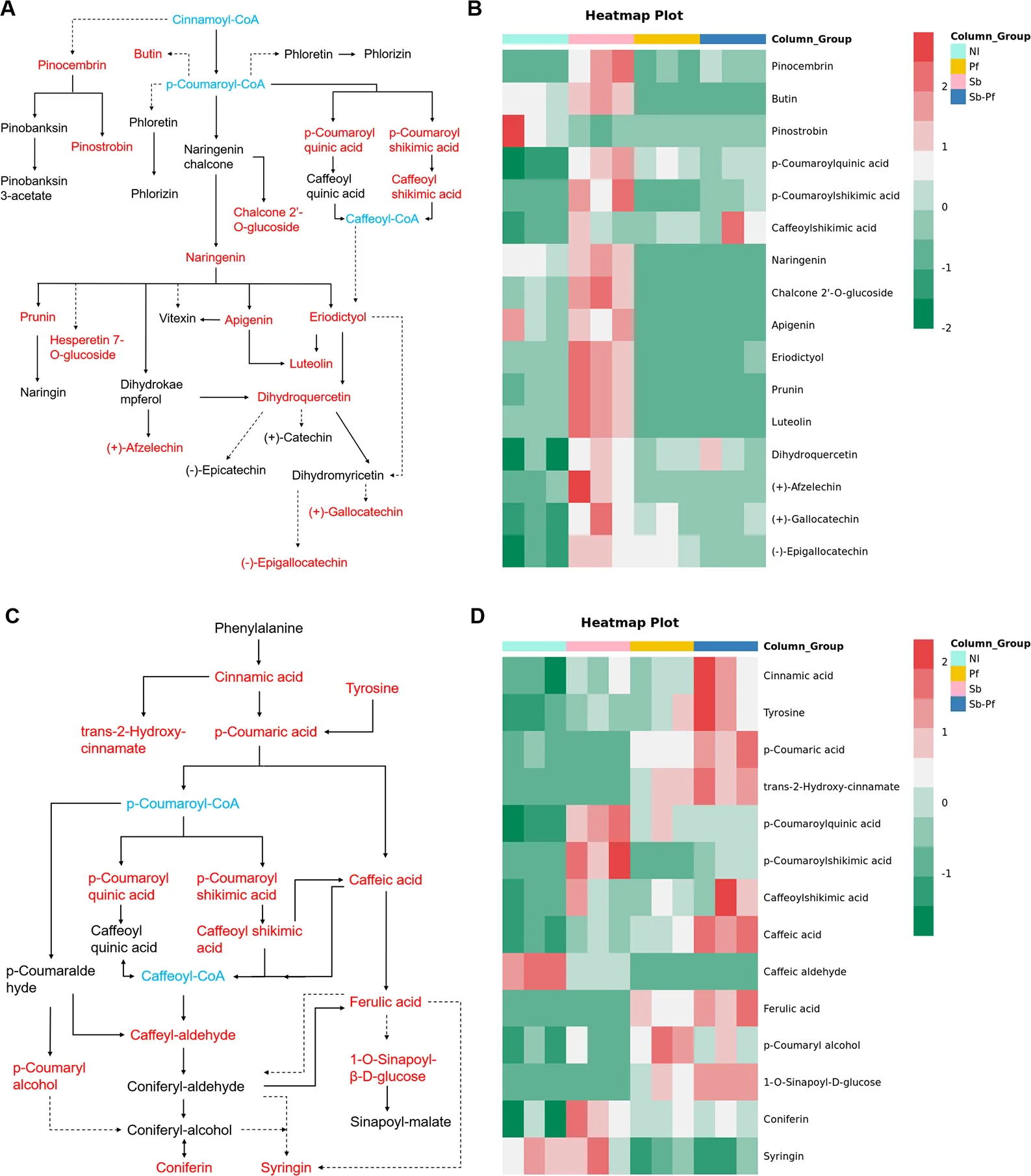
Flavonoid biosynthesis and phenylpropanoid biosynthesis pathways annotated with metabolites detected in the roots of *Pinus massoniana* seedlings. Seedlings either had not been inoculated (NI), inoculated with *Suillus bovinus* (Sb), inoculated with *Phialocephala fortinii* (Pf), or inoculated with both fungi (Sb–Pf). The backbones of the flavonoid biosynthesis (**A**) and phenylpropanoid biosynthesis (**C**) pathways are shown. Metabolites in red indicate upregulated metabolites; those in black showed no changes relative to the control. Metabolites in blue were not detected. Solid black arrows show the direct transformation of metabolites; broken arrows indicate that the transformation of metabolites involves at least two steps. Heatmap visualization of differentially accumulated metabolites annotated to the flavonoid biosynthesis (**B**) and phenylpropanoid biosynthesis (**D**) pathways. The content of each metabolite was normalized to complete linkage hierarchical clustering. Each example is visualized in a single column, and each metabolite is represented by a single row. Red indicates high abundance, whereas metabolites with a low relative abundance are shown in green.

KEGG (Kyoto Encyclopedia of Genes and Genomes) annotation revealed that DAMs synthesized under different treatments are involved in several different biological processes (Fig. 8B through D). DAMs produced under the Sb treatment are mainly involved in flavonoid biosynthesis, whereas DAMs produced under Pf or Sb–Pf treatments are mainly involved in phenylpropanoid biosynthesis. Among metabolites associated with the flavonoid biosynthesis pathway, approximately 55% of metabolites were upregulated under the Sb treatment (Fig. 9A). By contrast, DAMs associated with the phenylpropanoid biosynthesis pathway were mainly found in seedlings subjected to the Pf or Sb–Pf treatments (Fig. 9B).

## DISCUSSION

The prevalence of both ECM fungi and DSEs in roots of a wide spectrum of tree species is well recognized (2, 5); however, compared with functional studies, far less is known about the interactions that occur among ECM fungi, DSEs, and their co-host. In this study, we investigated interactions that occur among a typical dual symbiotic association formed by *S. bovinus* and *Phi. fortinii* and their co-host, *P. massoniana*, which is prevalent in forests of South China. Morphological and metabolic analyses revealed that these relationships were compatible. However, our findings may be specific to this particular combination of fungi and tree species. Furthermore, the lifestyles of root-colonizing fungi can vary, depending on the physiology of the plant and the availability of resources (32, 33).

### Asymbiotic co-culture of *S. bovinus* and *Phi. fortinii in vitro*

Interactions between ECM fungi and DSE may be species specific, given that synergistic, neutral, and antagonistic interactions have been found, depending on the pairs of ECM fungi and DSEs involved (4). In this study, neither antagonism nor promoting effects were observed when *Phi. fortinii* and *S. bovinus* were co-cultured *in vitro*. However, when colonies of these two fungi encountered each other, a spatial competition effect was observed, and more melanin accumulated in *Phi. fortinii* mycelium (Fig. 1; see also Fig. S2 in the supplemental material). DSEs are well-known for being melanin enriched, which may enhance their tolerance of variable adverse environments (34 – 36). Alternatively, the accumulation of melanin in *Phi. fortinii* may indicate a stress response due to spatial and perhaps also nutrient competition with *S. bovinus*. Moreover, the interactions observed when the two fungi were co-cultured *in vitro* may not fully reflect the interactions observed *in vivo*. Yakti et al. (37) reported similar findings: although DSE and pathogens exhibited mutual inhibition *in vitro*, this interactive effect was not observed when the host plant was inoculated with these fungi.

### Impact of *S. bovinus* and *Phi. fortinii* co-inoculation on each other’s establishment of a symbiotic association with *P. massoniana*

Although DSEs are ubiquitous in forest ecosystems, little is known about the mechanisms involved in the formation of a symbiotic association between these fungi and their hosts (6). In this study, we observed that the symbiotic association that developed between *Phi. fortinii* and *P. massoniana* differed from that of ECM. Generally, the symbiotic association between *Phi. fortinii* and *P. massoniana* was established more rapidly than the ECM formed by *S. bovinus* and *P. massoniana*. The *Phi. fortinii* functional structure—the microsclerotium—was intensively formed within the root at 8 dpi. By contrast, *S. bovinus* mycelium started to attach to the root surface at 7 dpi, and the functional structures—the mantle and Hartig net—formed at 28 dpi. Similar findings have also been reported in other studies (38 – 40). This pattern may be at least partially ascribed to the faster mycelial growth rate of *Phi. fortinii* compared with that of *S. bovinus*. Studies on fungal succession have described *Suillus* fungi as early-stage ECM fungi that rapidly react to hosts and play important roles in the establishment of pine invasions and advances (26, 28, 41). Considering the nutritional benefits as well as the functions of alleviating adverse conditions to host plants by DSEs (6, 7), our findings suggest that *Phi. fortinii* may play the same role or even a superior role to that of *S. bovinus* in *P. massoniana* establishment.

The symbiotic processes observed in this study are likely to be specific with respect to both fungal species and host plant species, and the time required for symbiotic structure formation differs between distinct fungal species and host plant combinations. Horan et al. (38) reported that the hyphae of *Paxillus involutus* and *Pisolithus tinctorius* can invade the root system of *Eucalyptus globulus* at 2 dpi and can establish ECM symbiotic associations with the formation of a mantle and a Hartig net in 2 weeks. Duplessis et al. (39) observed that the mycelium of *Pisolithus microcarpus* started to infect the roots of *E. globulus* at 4 dpi and formed mature mycorrhizae after 21 days. Similarly, Yu et al. (40) reported that *Phi. fortinii* mycelium began to invade the root system of *Asparagus officinalis* at 9 dpi; however, the formation of microsclerotia was observed only after 2 months. Moreover, the experimental system and culture environment may also affect the time needed for symbiont formation (42).

ECM fungi and DSEs are known to infect the root of the same host plant and to form multiple symbiotic associations (3). In this study, we also found that *S. bovinus* and *Phi. fortinii* were able to form a symbiotic relationship with the same *P. massoniana* seedling. Generally, the inoculation sequence did not affect the formation of these two symbiotic associations, indicating that a compatible relationship exists between these two symbionts. The inoculation sequence had only a slight influence on the morphogenesis of the symbiotic structures. The formation of the mantle lagged (the extraradical hyphae only loosely surrounding the root surface at 14 dpi) in the Pf + Sb treatment, and the Hartig net was less developed in the Sb–Pf treatment (Fig. 3). In both the Pf and Sb + Pf treatments, microsclerotia were mainly formed by hyaline mycelium, whereas in the Sb–Pf treatment, microsclerotia were formed by melanized mycelium (Fig. 4). These observations indicate that *S. bovinus* and *Phi. fortinii* interact when colonizing the root of the same host plant. Considering the *in vitro* co-culture observations as well as the essential roles of melanin in the regulation of various biological processes in the fungi and helping DSEs to get along with variable environments (35, 36, 43), we suggest that melanin may play an important role in helping *Phi. fortinii* to get along with *S. bovinus*. Further studies to elucidate the functions of melanin in these processes should increase our understanding of the interactions that occur between these two fungi.

**Fig 10:**
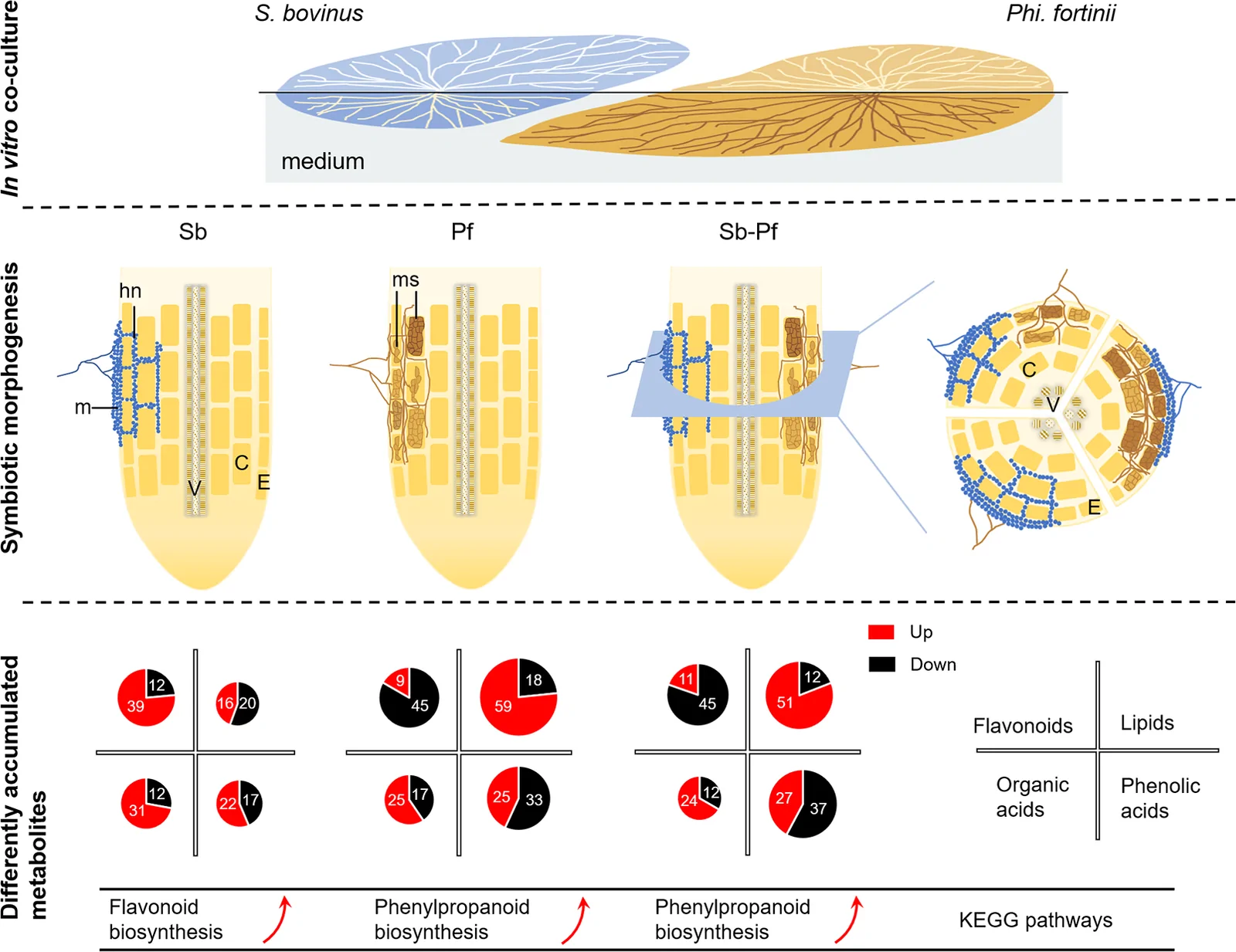
Schematic diagrams showing the interactions among *Suillus bovinus* (Sb), *Phialocephala fortinii* (Pf), and *Pinus massoniana in vitro* and *in vivo*, and differentially accumulated metabolites in roots. Seedlings had been either not inoculated (NI), inoculated with Sb, inoculated with Pf, or inoculated with both fungi (Sb–Pf). ‘C, E, V, hn, m, ms’ indicate cortical cells, epidermal cells, vascular system, Hartig net, mantle, and microsclerotia, respectively.

*S. bovinus* and *Phi. fortinii* were not only able to colonize the roots of the same seedling but also the same root segment locus (Fig. 5). When colonizing the same root locus (observed in root cross sections), there were generally three forms of colonization: the two fungi simultaneously colonized the root from different directions of the root periphery; *S. bovinus* colonized and formed a mantle around the root where *Phi. fortinii* had already colonized and formed microsclerotia; *Phi. fortinii* did not colonize the root where a physical barrier (i.e., the mantle) had already been formed by *S. bovinus* (Fig. 5 and 10). The first two types of colonization could explain why PAC species are found in ectomycorrhizal root tips occasionally (3, 18). However, based on our findings, it may be more appropriate to address that ECM fungi can colonize the root tips where DSEs have already invaded. The colonization of the lignified parts of tree roots by DSE is well-known, whereas ECM fungi mainly invade root tips (2). The reason for these distribution patterns in roots may be because ECM fungi tend to colonize root tips, and DSEs are unable to invade roots in which an ECM mantle has already formed (the third type of colonization). Overall, these results indicate that spatial competition occurs in the microniche between these two fungi and that *S. bovinus* appears to be superior to *Phi. fortinii* in this microniche competition.

### Effects of *S. bovinus* and/or *Phi. fortinii* inoculation on *P. massoniana* seedling growth

DSEs are thought to have an ecological function like that of mycorrhiza (44 – 46). In this study, we also found that the inoculation of *Phi. fortinii* significantly promoted the growth of *P. massoniana*, analogous to the effects of *S. bovinus* (Fig. 6). Similar findings have been reported elsewhere (22, 45, 47, 48). The co-inoculation treatment also promoted the growth of *P. massoniana*, but its promotion effect was weaker than the single-inoculation treatments. Previous studies have reported that culture conditions can have profound effects on the type of interactions that occur between DSEs and their hosts (13, 45, 49, 50). The inoculation effects observed in this study may be due to the ecological niche and nutrient competition between *S. bovinus* and *Phi. fortinii*, given that we used a closed experimental system with relatively limited space and nutrients.

Improved host nutrition [mainly nitrogen (N) and phosphorus (P)] due to nutrient uptake by ECM (2, 51, 52) and DSE (7, 53 – 55) has been well-addressed previously. However, in this study, we found that neither *S. bovinus* nor *Phi. fortinii* had any effect on N absorption, and even inhibited *P. massoniana* P accumulation. Astonishingly, fungal inoculation (particularly the dual-inoculation treatment) greatly promoted the potassium (K) accumulation of *P. massoniana* seedlings. Recent studies have shown that K can be transported through ECM fungi toward woody host plants and that the effects may be ECM fungal species specific (56 – 58). Peng et al. (58) reported that *S. bovinus* greatly promoted the K uptake of *P. massoniana*. The facultative ECM fungus *Clitopilus hobsonii* has been shown to largely promote the K uptake of *Liquidambar styraciflua*, whereas N and P uptake was inhibited (59). Similarly, Huang et al. (60) observed that *Tuber pseudohimalayense* and *Tuber formosanum* colonization enhanced the K uptake of some *Quercus* species but had no effect on their N and P uptake. However, there is little information on the role of DSEs in host plant K nutrition. Our analyses suggest that *Phi. fortinii* may play a role in host K uptake, although our observations may be at least partially ascribed to the relatively limited nutrients in the experimental system, particularly N and P. Furthermore, the extraradical fungal mycelium would have also taken up indispensable amounts of N and P, which were not considered in this study. In contrast, K was more abundant (4.62 mM compared with 1.25 mM of P in the Douglas-fir and sugar pine medium [DCR]) and more diffusible and, thus, was more available to both fungi and plants.

### Impact of *S. bovinus* and/or *Phi. fortinii* inoculation on metabolite accumulation in *P. massoniana* seedling roots

The inoculation of *S. bovinus* and/or *Phi. fortinii* affected the accumulation of metabolites in *P. massoniana* roots. Similar metabolic adjustments (i.e., the overall upregulation and downregulation of metabolites compared with NI treatment) were detected as a result of the *S. bovinus–P. massoniana* and *Phi. fortinii–P. massoniana* symbiotic associations, with a total of 152 shared DAMs found among all inoculation treatments, which were dominated by organic acids, lipids, phenolic acids, and flavonoids. These shared DAMs may play important roles in the synthesis and functionality of these symbioses and indicate that common metabolic adjustments exist between the two different symbiotic systems. Actually, there are already evidences that different symbiotic associations may have something in common, just like the common signaling pathway is involved in the promotion of both rhizobial and arbuscular mycorrhizal associations (61).

Generally, *S. bovinus* mainly affected flavonoid and organic acid accumulation, whereas *Phi. fortinii* greatly altered the accumulation of lipids and phenolic acids. Flavonoids can act as signaling molecules in plant–mycorrhizal fungi interactions to promote spore germination and mycelial growth (62, 63). Organic acids are common metabolites in plant roots, converting insoluble substances in the soil into effective nutrients that are conducive to plant absorption and utilization through processes such as acidification and chelation, as well as promoting nutrient absorption and growth of plants (64, 65). Some studies have reported that DSEs mostly use lipids as the storage form of energy substances (40). Given the large amount of lipids that had accumulated in the *Phi. fortinii* mycelium invading *P. massoniana* roots (Fig. S6) and the increased accumulation of lipids detected, we suggest that lipids may play vital roles in maintaining the DSE–host symbiotic relationship. Furthermore, host plants have been shown to transfer lipids to arbuscular mycorrhizal fungi to sustain the symbiosis (66). It seems likely that this also happens in the DSE–host symbiosis. Phenolic acids are involved in the cross linking of plant cell walls and are precursors of a variety of antimicrobial compounds, root signaling molecules, and phytoalexins, which play an important role in plant defense responses (67) and in plant–microbe interactions (68). Therefore, phenolic acids may play important roles in maintaining the *Phi. fortinii–P. massoniana* symbiotic association. Overall, to verify the functions of these DAMs, further research on the effects of *in vitro* application of the pure compounds, as well as the effects of genetic modifications of the DAM synthesis pathways, is needed.

*Phi. fortinii* had a greater impact on root metabolic adjustment than *S. bovinus*, given that the accumulation pattern of metabolites induced by the dual-inoculation treatment shared more DAMs with those induced by *Phi. fortinii* (Fig. 8A). The metabolic changes induced by the dual-inoculation treatment mimic those induced by *Phi. fortinii*, which may be at least partially ascribed to the faster growth and colonization processes of *Phi. fortinii* compared with those of *S. bovinus*. There were also 37 DAMs specifically detected in roots subjected to the Sb–Pf treatment, which may indicate that interaction effects occur between *S. bovinus* and *Phi. fortinii* when colonizing the same root system.

The accommodation of fungal symbionts within root tissues requires the coordination of a large number of plant metabolic and developmental pathways (17). KEGG pathway annotation revealed that DAMs detected in the different treatments are involved in several different biological processes (Fig. 8B through D). Similar results involving other ECM–host interactions have been observed in both transcriptional (11, 12, 16, 69) and metabolic (70, 71) studies. However, there is a lack of knowledge about other DSE–host associations. Among all the biological processes, the phenylpropanoid biosynthesis (Pf and Sb–Pf treatments) and the flavonoid biosynthesis (Sb treatment) pathways had the largest numbers of DAMs (Fig. 8B through D). These two pathways play important roles in regulating multiple plant developmental processes and signaling networks, and flavonoid biosynthesis originates from the phenylpropanoid pathway (the two pathways also share several metabolites) (72).

Flavonoids have long been recognized as signals that improve several plant–microbe interactions (such as rhizobia symbiosis, actinorhizal symbiosis, and arbuscular mycorrhizal symbiosis) (73 – 75), and our results (39 up-accumulated flavonoids were detected within roots of the Sb treatment) indicate that flavonoids may also play an important role in regulating symbiotic signaling in ECM. Flavonoids have been widely found to promote spore germination and hyphal ramification of ECM fungi (62, 63). Rutin, a flavonoid from *Eucalyptus globulus* subsp. *bicostata* root exudates, is considered a signal for *Pisolithus*, which may further promote the ECM formation (76). Furthermore, a *Suillus* species—*Suillus variegatus*—has been shown to influence the accumulation of flavonoids by its host plant—*Pinus sylvestris* (70).

The phenylpropanoid pathway has multiple functions, and its end products (such as lignins) are involved in plant defense responses. Furthermore, previous studies have reported that DSE colonization can induce temporally plant defense responses (40, 77, 78). The upregulation of the phenylpropanoid pathway may indicate the still ongoing symbiotic process between *Phi. fortinii* and *P. massoniana*. Furthermore, there was a higher concentration of methyl jasmonate in *P. massoniana* roots inoculated with *Phi. fortinii* (Pf and Sb–Pf treatments) than in those subjected to NI or Sb treatments. Methyl jasmonate is a hormone that mediates diverse developmental processes and defense responses against biotic and abiotic stresses (79, 80). This finding further indicates that DSE colonization involved the coordination of plant defense responses. By contrast, the defense-­like responses in host roots that have been colonized by ECM fungi are impaired (17, 81, 82) and usually happen at the early stage (several days to 1 or 2 weeks post ECM fungal inoculation) of ECM formation (11, 12, 16, 69, 71).

### Conclusion

In conclusion, our morphological and metabolic analyses show that *S. bovinus* and *Phi. fortinii* form a compatible relationship with their co-host, *P. massoniana* (Fig. 10). There were no synergistic or antagonistic effects between *Phi. fortinii* and *S. bovinus* when co-cultured. Furthermore, *S. bovinus* and *Phi. fortinii* co-inoculation did not affect each other’s formation of a symbiotic association with *P. massoniana* roots. Competition occurred when the two fungi colonized the same locus of a root: *S. bovinus* was able to colonize the root locus where *Phi. fortinii* had already invaded but not vice versa. Both *S. bovinus* and *Phi. fortinii* significantly promoted the growth of *P. massoniana* and altered the accumulation patterns of root metabolites, especially organic acids, flavonoids, lipids, and phenolic acids. *S. bovinus* inoculation significantly enhanced the flavonoid biosynthesis pathway, whereas *Phi. fortinii* altered the phenylpropanoid biosynthesis pathway. Our findings provide a theoretical basis for the co-application of these two prevalent symbiotic fungi when cultivating *P. massoniana* seedlings and highlight the need for an in-depth investigation of the roles of the two pathways in the symbiotic process between fungi and plant roots.

## MATERIALS AND METHODS

### Biological materials

Fungal strains *Phialocephala fortinii* LL-Pm-1 (24) and *Suillus bovinus* LL-Ps-001 (22, 23) were previously isolated from *Pinus massoniana* root and sporocarp, respectively. The stains were previously identified, combining both morphological and molecular methods (the accession numbers of ITS sequences deposited in GenBank database were MH681581 and MN888752, respectively). Seeds of *P. massoniana* were collected at the Ma’anshan Forest Farm in Duyun, Guizhou Province (P.R. China).

### Experiment 1: interactions between *S. bovinus* and *Phi. fortinii in vitro*

Prior to the start of the experiment, the strains were grown on potato dextrose agar medium for 2 weeks at 25°C in the dark. To investigate interactions between the two fungal strains, a co-culture experiment was performed according to Berthelot et al. (4). Plates of PDA medium were inoculated with a plug (8 mm in diameter) of each of the two fungi. Plugs were placed 4 cm apart from each other (10 plates were made; i.e., *n* = 10). The controls consisted of two plugs of the same strain (*n* = 10).

Colony growth was recorded at 12 dpi. The internal-to-external radii ratio and colony diameter (*D*) were recorded. When the hyphae from the two colonies made contact (20 dpi for *Phi. fortinii* control plates, 20 dpi for co-cultures of *S. bovinus* and *Phi. fortinii*, and 30 dpi for *S. bovinus* control plates), mycelia in the intersection zones were observed under a stereo microscope (Leica, M205FA).

### Experiment 2: impact of *S. bovinus* and *Phi. fortinii* co-inoculation on each other’s establishment of a symbiotic association with *P. massoniana*

#### Experimental setup

The experiment was conducted following the procedures described by Feng et al. (22). *P. massoniana* seeds were surface sterilized with 0.5% KMnO_4_ + 0.01% Tween 20 for 2 h before being washed three times with sterilized water, followed by 0.5% carbendazol + 0.01% Tween 20 for 1 h before being washed three times with sterilized water, followed by further sterilization with an antibiotic solution (200 mg/L streptomycin and 100 mg/L gentamycin) for 15 min before being washed three times with sterilized water. Sterilized seeds were left to germinate in water agar medium (0.8%) at 25°C in the dark for 10 days. Germinated seedlings were then exposed to light for 20 days prior to the start of the inoculation trials.

Three seedlings were transferred to a square petri dish (13 cm × 13 cm) containing 25 mL of DCR medium, and fungal plugs (1 cm in diameter) from a 2-week-old culture were placed approximately 2 mm away from the lateral roots. Petri dishes were sealed with Parafilm and partially wrapped with aluminum-foil paper to cover the part where the root grew. Petri dishes were placed vertically in a growth chamber with a 14-h light (light 150 µmol/m^2^/s)/10-h dark (light 0 µmol/m^2^/s) cycle at 25°C.

The experiment involved five treatments: (i) a single inoculation of *S. bovinus*, (ii) a single inoculation of *Phi. fortinii*, (iii) a single inoculation of *S. bovinus* followed by inoculation with *Phi. fortinii* after 28 days (Sb + Pf) [based on a previous study showing that *S. bovinus* can colonize a *P. massoniana* root and form a typical mantle and Hartig net in 30 days; (22)], (iv) a single inoculation of *Phi. fortinii* followed by inoculation with *S. bovinus* after 7 days (Pf + Sb) [based on our previous research (24)], and (v) simultaneous inoculation of *S. bovinus* and *Phi. fortinii* (Sb–Pf) (Fig. S7). There were 20 plates of each treatment.

#### Morphological observations

Root samples were collected regularly to observe the formation of symbiotic associations: the formation of ectomycorrhizas was checked every 7 days, and the formation of a symbiotic association between *Phi. fortinii* and *P. massoniana* was checked every 2 days. Root samples were observed under a stereo microscope (Leica, M205FA) to check for the presence of extraradical mycelium, and microstructures (i.e., the formation of a mantle and Hartig net by *S. bovinus* or microsclerotia by *Phi. fortinii*) were observed using a compound microscope (Leica, DM3000) after roots were hand-sliced and stained with 0.05% trypan blue (22).

To better distinguish the microstructures formed by the different types of fungi, scanning electron microscopy was used to observe the formation of each symbiont in the single-inoculation treatments. Root samples were hand-sliced, fixed in 2.5% glutaraldehyde at 4°C overnight, and then washed with 0.2 M PBS (phosphate buffered saline) solution (pH 7.2) three times (15 min each time). The slices were then treated with 35%, 50%, and 75% ethanol for 30 min, followed by immersion in 95% and 100% ethanol twice (30 min each time). After, the slices were further treated with a 3:1, 2:2, and 1:3 (vol:vol) ethanol and tert-butanol mixture (each lasting 10 min) and were finally treated with 100% tert-butanol for 10 min. The tert-butanol was removed from root samples by freeze-drying under vacuum. The dehydrated root slices were stuck to conductive tape on a specimen holder before coating the holder with Au on a rotating specimen stage (45 s). Samples were then observed under a scanning electron microscope (TM4000plus; Hitachi) (voltage, 15 kV; degree of vacuum, high vacuum; electronic detection mode, backscattered electrons).

### Experiment 3: impact of *S. bovinus* and/or *Phi. fortinii* inoculation on the *P. massoniana* root metabolome

#### Experimental setup

Fungal inocula and *P. massoniana* seedlings were prepared and cultured as above. Seedlings were inoculated with either *S. bovinus*, *Phi. fortinii*, or both *S. bovinus* and *Phi. fortinii* (Sb–Pf), or were not inoculated (NI) (30 plates of each treatment). Seedlings were cultured under a 14-h light (intensity 150 µmol/m^2^/s)/10 h dark cycle at 25°C.

#### Sampling and N, P, and K determination

At 60 dpi, the seedlings were harvested. The needles were counted and the fresh weight of the seedling was recorded (one seedling makes one sample, and five seedlings of each treatment were checked; i.e., *n* = 5). A scanner (Epson Perfection V330Photo) was used to collect images of the roots. The SmartRoot plugin of the ImageJ platform was used to determine the root length and the number of root tips (*n* = 5).

To determine the nitrogen (N), phosphorus (P), and potassium (K) concentrations, plant samples (whole plants) were harvested and dried at 65°C to a constant weight. Dried seedlings, including roots, stems, and leaves, were ground into powder using an oscillating grinder. The Kjeldahl digestion procedure was used for total N determination (83). P concentration was quantified by the Mo-Sb colorimetric method after digestion with H_2_SO_4_–H_2_O_2_ (84). K^+^ determination was performed using the inductively coupled plasma-atomic emission spectrometry (ICP-AES) system of iCAP 6300 (Thermo Fisher Scientific Inc., San Jose, CA, USA). There were five biological replicates of each of these metrics.

Roots of the remaining seedlings in each treatment were collected and combined to make three independent samples of approximately 1 g (fresh weight). After freeze-drying using a vacuum freeze-dryer (Scientz-100F), root samples were stored at –80°C until metabolome analysis.

#### Metabolite extraction and analysis

Root samples were crushed using a mixer mill (MM 400; Retsch) with zirconia beads for 1.5 min at 30 Hz. Next, 100 mg of the lyophilized powder sample was dissolved with 1.2 mL of 70% methanol solution, vortexed for 30 s every 30 min six times, and then placed in a refrigerator at 4°C overnight. Following centrifugation at 12,000 rpm for 10 min, extracts were filtrated (SCAA-104, 0.22-µm pore size; ANPEL, Shanghai, China, http://www.anpel.com.cn/) before liquid chromatography–mass spectrometry analysis.

Sample extracts were analyzed using an ultra-high-performance liquid chromatography (UPLC)–electrospray ionization (ESI)–tandem mass spectrometry (MS/MS) system (UPLC, SHIMADZU Nexera X2, www.shimadzu.com.cn; MS, Applied Biosystems 4500 Q TRAP, www.appliedbiosystems.com.cn/). Analytical conditions were as follows: UPLC, column, Agilent SB-C18 (1.8 µm, 2.1 mm × 100 mm); a mobile phase comprising solvent A, pure water with 0.1% formic acid, and solvent B, acetonitrile with 0.1% formic acid. Sample measurements were performed using a gradient program with the following starting conditions: 95% A and 5% B. Within 9 min, a linear gradient to 5% A and 95% B was programmed, and a composition of 5% A and 95% B was maintained for 1 min. Within 1.1 min, the composition was adjusted to 95% A and 5% B, which was maintained for 2.9 min. The flow velocity was set at 0.35 mL per minute; the column oven was set to 40°C; the injection volume was 4 µL. The effluent was connected to an ESI–triple quadrupole-linear ion trap (QTRAP)-mass spectrometry (MS).

Linear ion trap and triple quadrupole (QQQ) scans were acquired using a QTRAP-MS, API6500 QTRAP LC/MS/MS System, equipped with an ESI Turbo Ion-Spray interface, operating in positive ion mode, and controlled by Analyst v.1.6.3 software (AB Sciex). The ESI source operation parameters were as follows: ion source, turbo spray; source temperature, 500°C; ion spray voltage, 5,500 V; ion source, gas I, gas II, and curtain gas were set at 55, 60, and 25.0 psi, respectively; collision gas pressure, high. Instrument tuning and mass calibration were performed with 10 and 100 µmol/L polypropylene glycol solutions in QQQ and linear ion trap modes, respectively. QQQ scans were acquired as multiple reaction monitoring experiments with the collision gas (N) set to 5 psi. The declustering potential and collision energy for individual multiple reaction monitoring transitions were set with further declustering potential and collision energy optimization. A specific set of multiple reaction monitoring transitions was monitored for each period according to the metabolites eluted within this period.

Differentially accumulated metabolites (DAMs) were screened by supervised orthogonal projection to latent structure discriminant analysis according to the default criteria of fold change being ≥2 or ≤0.5, and the variable importance in project being ≥1 between LP and HP treatments. Each treatment had three biological replicates.

Identified metabolites were annotated using the KEGG Compound database (http://www.kegg.jp/kegg/compound/), and annotated metabolites were then mapped to the KEGG Pathway database (http://www.kegg.jp/kegg/pathway.html). Pathways with significantly regulated metabolites mapped to them were then analyzed using metabolite set enrichment analysis; their significance was determined by calculating the *P* values of a hypergeometric test.

### Data analysis

Mass spectrometry data were processed using Analyst v.1.6.3 software (AB Sciex Pte. Ltd., https://sciex.com/), and R software (The R Foundation, https://www.r-project.org/) was used for multivariate statistical analysis of the metabolic data. SPSS v.19.0.0 (SPSS Inc., http://www.spss.com.cn) was used for statistical analysis of morphology and nutrient content data, and a Student–Newman–Keuls *q* test was used for multiple comparisons.

## Data Availability

The data sets of metabolomes presented in this study are listed in Table S2. Other data are available from the corresponding author upon reasonable request.

## SUPPLEMENTAL MATERIAL

Fig. S1 to S7 spectrum.01453-23-s0001.docx Supplemental material showing the experiment setup, the morphological features of both seedlings and symbiotic structures, etc.

Table S1 spectrum.01453-23-s0002.docx Impact of *Suillus bovinus* and *Phialocephala fortinii* co-culture on colony growth after 12 days of co-culture.

Table S2 spectrum.01453-23-s0003.xlsx Metabolic profiles of *Pinus massoniana* roots.

Table S3 spectrum.01453-23-s0004.xlsx Fungal-induced differentially accumulated metabolites in *Pinus massoniana* roots.

ASM does not own the copyrights to Supplemental Material that may be linked to, or accessed through, an article. The authors have granted ASM a non-exclusive, world-wide license to publish the Supplemental Material files. Please contact the corresponding author directly for reuse.
