# Supplementary material for: Morphogenesis and metabolomics reveal the compatible relationship among Suillus bovinus, Phialocephala fortinii, and their co-host, Pinus massoniana
Source: Microbiol Spectr. 2023 Sep 7;11(5):e01453-23. doi: 10.1128/spectrum.01453-23 (PMC10580909; doi:10.1128/spectrum.01453-23)

**SUPPLEMENTAL FIGURES:**

**FIG S1** Mycelium of *Suillus bovinus* and *Phialocephala fortinii* colonies that have made contact. (A) Aerial mycelium; (B) substrate mycelium. Pf indicates the *Phi. fortinii* colony; Sb indicates the *S. bovinus* colony; blue stars indicate *Phi. fortinii* substrate mycelium; scale bars = 2 mm.


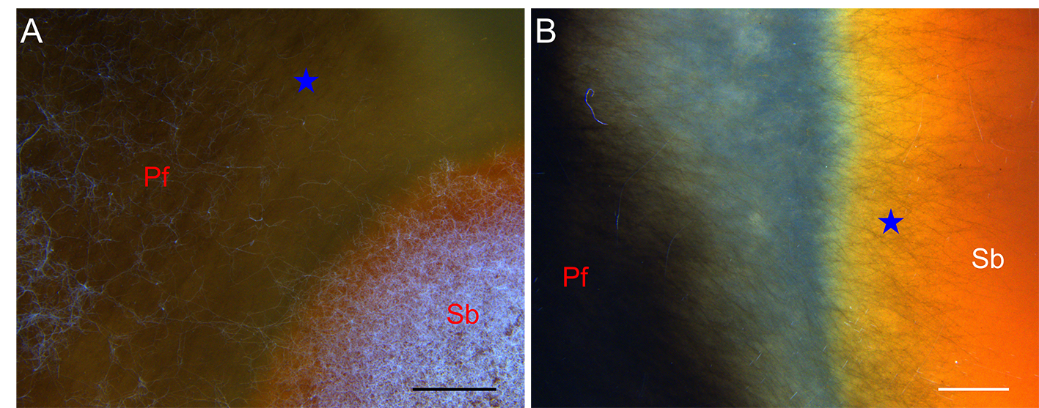


**FIG S2** Extraradical mycelium attached to the root surface of *Pinus massoniana* roots inoculated with a single fungal species. (A), (C), and (E) *Suillus bovinus* at 7, 14, and 28 days post inoculation (dpi), respectively. (B) and (D) Hyaline mycelium of *Phialocephala fortinii* at 2 and 4 dpi, respectively. (F) Melanized mycelium of *Phi. fortinii* attached to the root surface at 8 dpi. Scale bars = 2 mm.


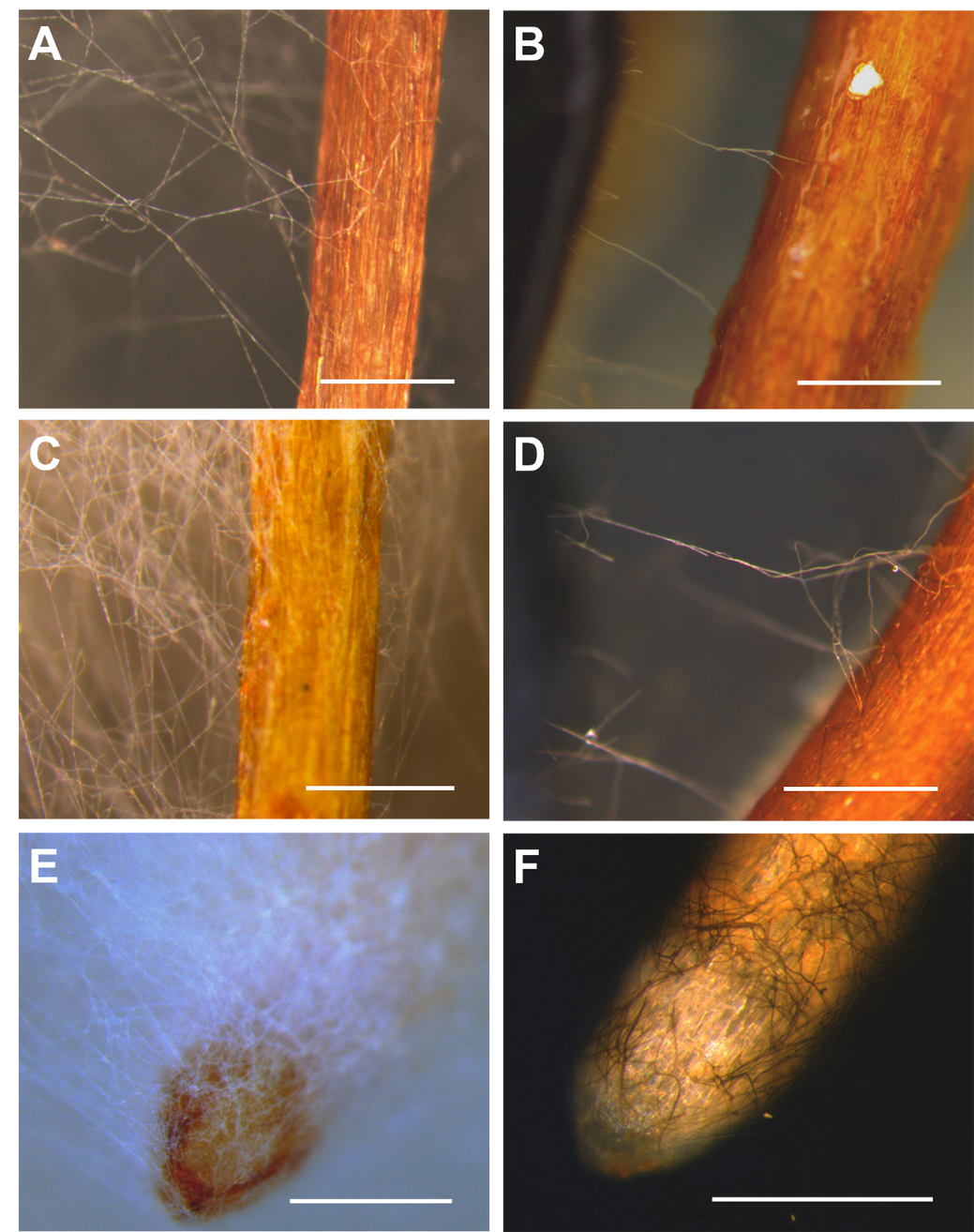


**FIG S3** Mantle and Hartig net formed by *Suillus bovinus* associated with a *Pinus massoniana* root at 28 days post inoculation (dpi) (A,B). Microsclerotia (M) formed by *Phialocephala fortinii* at 8 dpi (C,D). White arrowheads indicate the Hartig net; black arrowheads indicate the mantle. Scale bars = 50 μm.


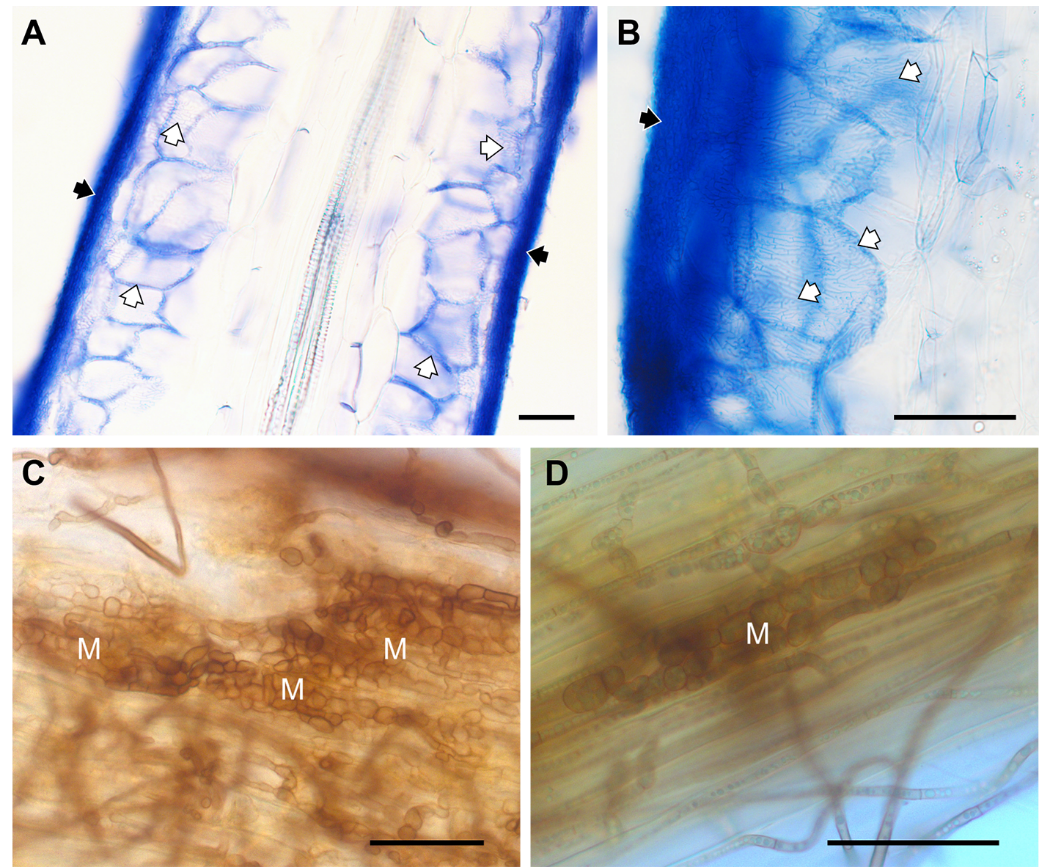


**FIG S4** *Pinus massoniana* seedlings that had been inoculated with: (A) *Phialocephala fortinii* (Pf), (B) *Suillus bovinus* (Sb), (C) both fungi (Sb–Pf), or (D) not inoculated (Ni) (28 dpi). Scale bars = 1 cm.


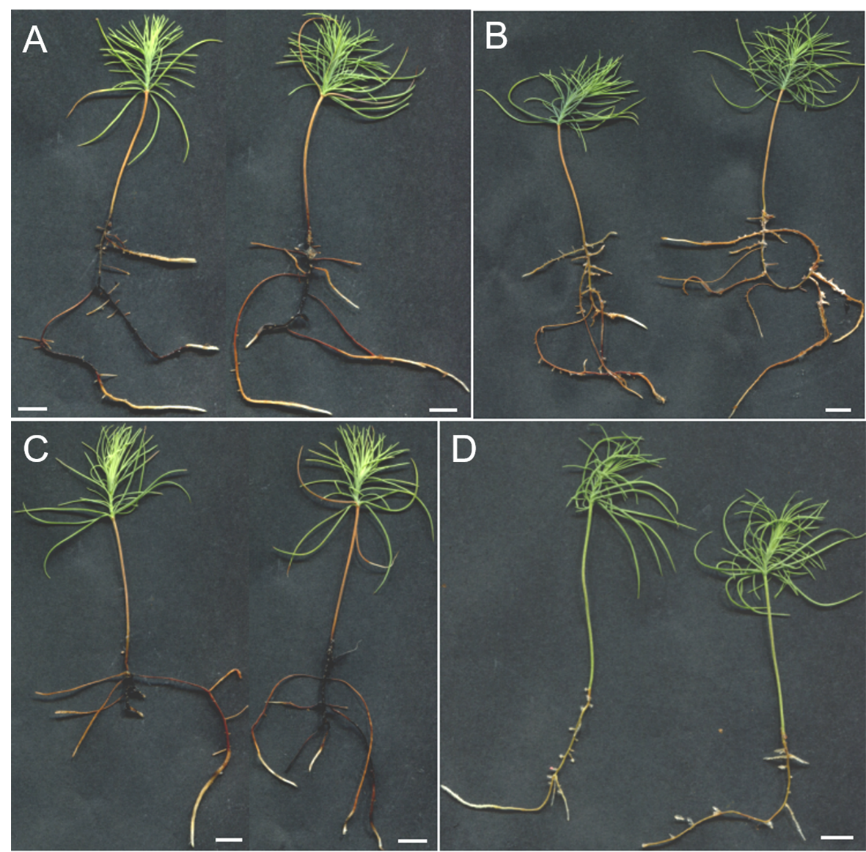


**FIG S5** Kmeans clusters of differentially accumulated metabolites. The ordinate represents the normalized relative metabolite abundance, and the subclass indicates the category and the number of metabolites showing the same accumulation patterns in the roots of *Pinus massoniana* seedlings that were either subjected to inoculation with *Suillus bovinus* (Sb), *Phialocephala fortinii* (Pf), or both fungi (Sb–Pf), or were not inoculated (Ni).


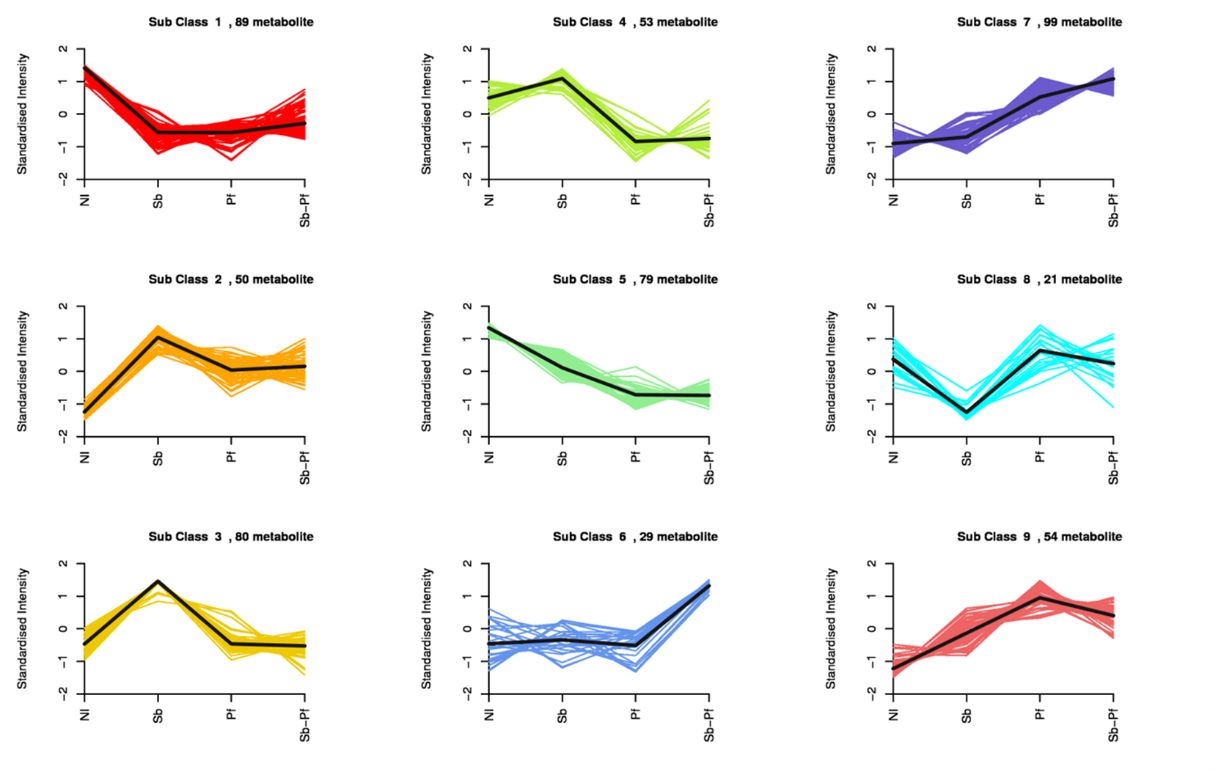


**FIG S6** Lipids deposits within the root of *Pinus massoniana*. (A) Root section of a non-inoculated seedling. The enlarged inset in the upper-right corner shows cells without lipid deposits. (B) Root section of a seedling that had been inoculated with *Phialocephala fortinii* showing lipid deposits in the cells. The lipid deposits have been stained red with Sudan III solution. The enlarged inset in the upper-right corner shows a microsclerotia-forming cell with lipids deposits. Scale bar = 100 μm.


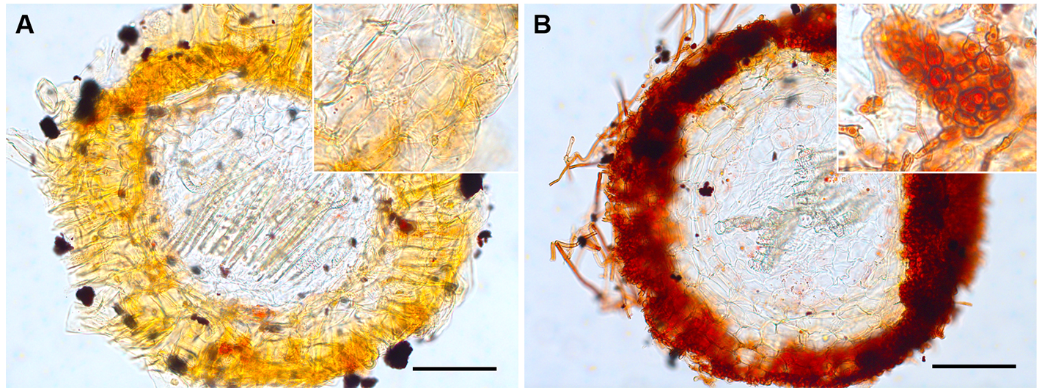


**FIG S7** Experimental setup used for symbiont morphogenesis analysis. (A) *Pinus massoniana* seedling roots inoculated with *Phialocephala fortinii* at 8 days post inoculation (dpi); (B) *P. massoniana* roots inoculated with *Suillus bovinus* at 28 dpi; (C) dual inoculated roots of *P. massoniana* 2 dpi with *Phi. fortinii* and 30 dpi with *S. bovinus*; (D) dual inoculated roots of *P. massoniana* 7 dpi with *S. bovinus* and 15 dpi with *Phi. fortinii*; (E) dual inoculated roots of *P. massoniana* at 8 dpi with *S. bovinus* and *Phi. fortinii*.


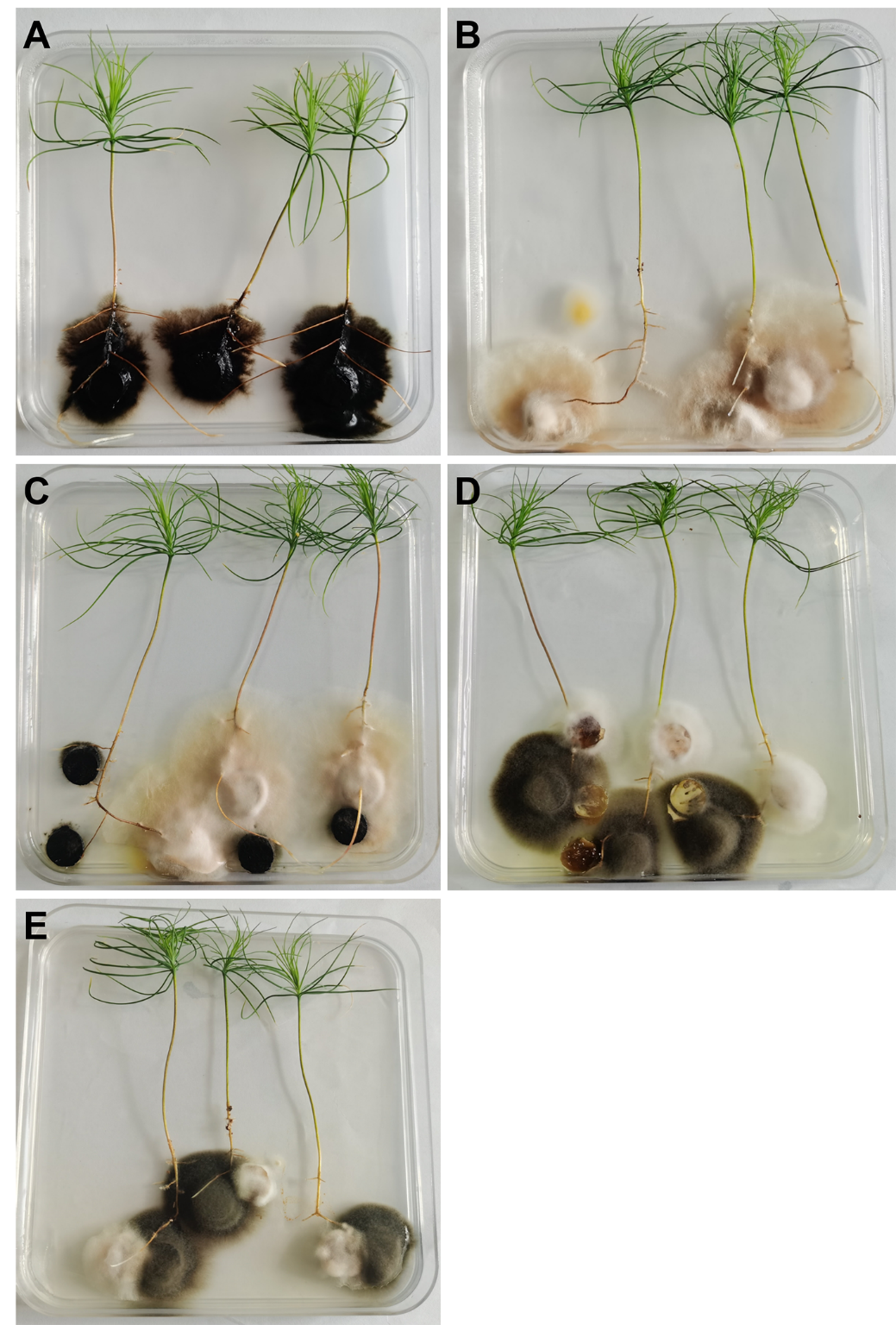

Supplement: Fig. S1 to S7 — Supplemental material showing the experiment setup, the morphological features of both seedlings and symbiotic structures, etc. [file spectrum.01453-23-s0001.docx]
