# Supplementary material for: Morphogenesis and metabolomics reveal the compatible relationship among Suillus bovinus, Phialocephala fortinii, and their co-host, Pinus massoniana
Source: Microbiol Spectr. 2023 Sep 7;11(5):e01453-23. doi: 10.1128/spectrum.01453-23 (PMC10580909; doi:10.1128/spectrum.01453-23)
Supplement: Table S1 — Impact of Suillus bovinus and Phialocephala fortinii co-culture on colony growth after 12 days of co-culture. [file spectrum.01453-23-s0002.docx]

**TABLE S1** Impact of *Suillus bovinus* and *Phialocephala fortinii* co-culture on colony growth after 12 d of co-culture

| Treatments | Diameter (cm) | Radii ratio (IR/ER) |
| --- | --- | --- |
| Sb–Pf (Pf) | 3.15 ± 0.10a | 1.00 ± 0.00a |
| Sb–Pf (Sb) | 1.51 ± 0.25b | 0.95 ± 0.04a |
| Pf–Pf | 3.02 ± 0.14a | 0.98 ± 0.03a |
| Sb–Sb | 1.64 ± 0.16b | 1.01 ± 0.16a |

Note: Sb–Pf (Pf) indicates the growth of *Phi. fortinii* colonies in the *S. bovinus* and *Phi. fortinii* co-culture treatment*.* Sb–Pf (Sb) indicates the growth of *S. bovinus* colonies in the *S. bovinus* and *Phi. fortinii* co-culture treatment*.* Pf–Pf indicates the growth of *Phi. fortinii* colonies in the single culture treatment (control). Sb–Sb indicates *S. bovinus* colonies in the single culture treatment (control)*.* IR/ER, internal and external colony radii ratio. Different lowercase letters indicate a significant difference between data in the same column, *P* < 0.05. *n* = 10.
